# Caballero–Engel meet Lasry–Lions: A uniqueness result

**DOI:** 10.1007/s11579-024-00370-2

**Published:** 2024-07-26

**Authors:** Fernando Alvarez, Francesco Lippi, Panagiotis Souganidis

**Affiliations:** 1 University of Chicago and NBER, Chicago, USA; 2 LUISS University and EIEF, Rome, Italy; 3 University of Chicago, Chicago, USA

**Keywords:** Mean field game, Impulse control, Dynamic games, C02, E10

## Abstract

In a Mean Field Game (MFG) each decision maker cares about the cross sectional distribution of the state and the dynamics of the distribution is generated by the agents’ optimal decisions. We prove the uniqueness of the equilibrium in a class of MFG where the decision maker controls the state at optimally chosen times. This setup accommodates several problems featuring non-convex adjustment costs, and complements the well known drift-control case studied by Lasry–Lions. Examples of such problems are described by Caballero and Engel in several papers, which introduce the concept of the generalized hazard function of adjustment. We extend the analysis to a general “impulse control problem” by introducing the concept of the “Impulse Hamiltonian”. Under the monotonicity assumption (a form of strategic substitutability), we establish the uniqueness of equilibrium. In this context, the Impulse Hamiltonian and its derivative play a similar role to the classical Hamiltonian that arises in the drift-control case.

## Introduction

Several interesting economic problems feature a continuum of agents subject to idiosyncratic shocks and aggregate dynamics. The equilibrium of these economies is a fixed point in which individual decisions depend on aggregates, and aggregate dynamics depend on individual decisions. The feedback between decisions and aggregates raises non-trivial questions about the uniqueness of the equilibrium which cannot be addressed computationally. Such fixed point problems can be represented by the mathematical structure of Mean Field Games (MFG), namely two coupled partial differential equations with appropriate boundary conditions: a Hamilton–Jacobi–Bellman equation, describing individual choices, and a Kolmogorov equation, describing the dynamics of the cross sectional distribution. The MFG formulation provides a rigorous definition of the problem which is useful to analyze the uniqueness of the equilibrium.

For a broad class of MFG problems, in which the decision maker controls *the drift* of a diffusion process, Lasry and Lions [18] establish a well-known uniqueness result for the equilibrium of a dynamic game given an arbitrary initial condition. These problems are akin to e.g., dynamic savings problems, as described by Aiyagari [2] and analyzed as a MFG by Achdou et al. [1]. An important feature of the Lasry–Lions result is that the uniqueness is ensured by a property of the flow cost function, the so called Lasry–Lions “monotonicity condition”. This property is a form of “strategic substitutability” of the players’ actions in a static game.

In this paper we present a complementary uniqueness result for a class of problems where the decision maker controls the stopping times for resetting the state, rather than controlling the drift. In other words, we examine the uniqueness of the equilibrium in a MFG characterized by a form of “impulse control” instead of “drift control”. These problems are akin to e.g., optimal investment or price-setting problems in the presence of fixed costs, pioneered by Caballero and Engel [10, 12]. Our setup assumes that the uncontrolled state follows a diffusion process, and that the decision maker can take (costly) actions that affect the probability to control the state. When such a control opportunity arises, the decision maker can reset the state to any desired level. Consequently, the state follows a diffusion process with jumps, and the intensity and size of these jumps are optimally determined.^1^ We demonstrate that under standard monotonicity conditions the MFG possesses a unique classical equilibrium.

Optimal decisions in the MFG with impulse control are described by two objects. The first is the optimal reset function, which represents the level where the state is located after an adjustment. The second is the probability per unit of time of an adjustment opportunity, which is also a function of the state and time. These two objects depend on time because the cross sectional distribution evolves through time, and the decision maker is forward looking. Equivalently, using the terms introduced by Caballero and Engel, the “optimal reset point” and the “generalized hazard rate” are time dependent and endogenous. The time dependence is due to the fact that the cross sectional distribution is time varying. The endogeneity is a consequence of the dependence of the cross sectional distribution on the optimal decision rules.

To analyze the problem described above we introduce a function which we label Impulse Hamiltonian. The Impulse Hamiltonian gives the optimized expected reduction in the cost, as a function of the difference between the value function at the current time and state and the minimized value function at the current time. The Impulse Hamiltonian serves a similar purpose as the Hamiltonian in the case of drift control. The Impulse Hamiltonian has convexity properties that are analogous to the ones of the standard Hamiltonian. Moreover, the Impulse Hamiltonian has the property, analogue to the one in the drift control, that its derivative gives the drift of the diffusion that appears in the Kolmogorov–Fokker–Planck equation. In particular, in the case of the Impulse Hamiltonian, the probability per unit of time of a change of the state equals (the negative) of its derivative. It turns out that, as in the drift-control case, the coupling of the Hamilton–Jacobi–Bellman equation with the Kolmogorov–Fokker–Planck equation can be done using the Impulse Hamiltonian and its derivative.

Our main result gives sufficient conditions for the equilibrium uniqueness of the MFG. The conditions are similar to the ones in the drift control case, and essentially require the “monotonicity” of the flow cost and terminal value function and some uniformity in the convexity of the Hamiltonian. Recall that the monotonicity of the period cost function has the interpretation of strategic substitutability in a static game. In our case, besides the same monotonicity condition on the period cost function, we require some uniformity on the first and second derivatives of the Impulse Hamiltonian. We can translate the conditions of the Impulse Hamiltonian to the distribution of random adjustment cost. For instance, the uniformity of the first derivative is equivalent to having a mass point on the zero cost of adjustment, which in economic models translate into having some adjustment as in the Calvo model, or in the Calvo-plus model. The uniformity of the second derivative is equivalent to a density of the fixed cost bounded from below.

We first illustrate the uniqueness result using a MFG without aggregate uncertainty, i.e., in the language of MFG, the case with “no aggregate noise”. We then consider two extensions. The first one adds an aggregate noise as often occurs in many applications in economics. The second one is a version where the controlled state is multidimensional. Examples of multidimensional control are the multiproduct pricing models of Midrigan [21], Alvarez and Lippi [4] and Bhattarai and Schoenle [8].

### Applications

We outline three decision problems that fit within our formulation. In the first problem the decision maker draws a fixed cost of adjustment from an arbitrary distribution with a constant probability per unit of time.^2^ If the decision maker pays the fixed cost she can exercise control and reset the state to any desired value. Otherwise the state of the agent remains uncontrolled and follows a diffusion process until the next (random) time when the decision maker has the opportunity to change the state. This type of decision problem was first proposed in Caballero and Engel [12], analyzed in Caballero and Engel [10, 14], and recently further characterized in Alvarez et al. [5], and has been used to study optimal investment and pricing decisions by firms. In these problems the decision maker selects a *time invariant probability of adjustment*, referred to as the *generalized hazard function*, as a function of the state. Additionally, upon adjusting, the state is optimally reset to a time-invariant value, known as the “reset point”.

The second problem stems from papers in applied mathematics that consider continuous-time setups where the decision maker controls a diffusion process only at random Poisson times. Some notable contributions in this area include Wang [22], Dupuis and Wang [17], and Menaldi and Robin [20].^3^ These papers develop the appropriate variational inequality that applies to the decision maker’s value function.

The third type of problems are economic models such as the one in Woodford [23] and Costain and Nakov [16], where agents choose a probability of adjustment per unit of time subject to a cost that increases with this probability.

We emphasize that the three problems outlined above consider a decision maker facing a time-invariant environment, i.e., a stationary distribution of the state. In these environments there is no feedback from the aggregate state to individual decisions.^4^ Instead, in this paper we consider such decision problems as part of a MFG, by focusing on setups where the distribution of the state evolves dynamically in a way that depends on the agents’ decisions. In particular, we analyze the uniqueness of the MFG equilibrium, i.e., the uniqueness of the fixed point, where the decision maker’s flow cost depends on the cross-sectional distribution, and the cross-sectional distribution evolves according to the optimal decisions of agents.

Our contributions relate to the literature in MFGs. In particular, there are many papers on uniqueness on MFGs based on the seminal contribution by Lasry and Lions [18] and the literature that follows it. The vast majority of this literature focuses on the case of drift control, and has extended their initial results to different set ups. Our work is related to Bertucci [7] and Alvarez et al. [3]. These two papers, and the current one, share the property that agents choose when to pay a fixed cost to change the value of the state. As a consequence, the optimal decision rule at each time has the form of dividing the state space in two regions, one where there is inaction, and one where control is exercised, i.e., for a given state adjustment either occurs or not. But Bertucci [7] differs from Alvarez et al. [3] and the current paper because, when stopping, the agent does not reset the value at a certain “location”, i.e., the min value. As a result the density is “not preserved”, that is some players choose to leave the game. Instead, in Alvarez et al. [3] and the current paper the agents choose the optimal adjustment rate, which varies with the value of the state, and never leave the game so that the total density is preserved. More differences need to be noted: in Alvarez et al. [3] we consider only a perturbation of the MFG equilibrium, but we allow both for the case of monotonicity and “anti-monotonicity”, i.e., strategic substitutability and complementarity.

We finish with a remark on the separability assumption used in the period cost function. In our specification we follow the simplest and most widely used specification where the cost function has an additive separable structure. In particular, there is additive separability in the period cost function between the effect of (*x*, *m*), i.e., the state and the cross sectional density, and the state *x* and the cost of adjustment. This is the same type of separability often assumed in drift control MFGs. The assumption is important since it allows a simple version of the monotonicity conditions. In several drift control problems, such as in the Aiyagari [2] model, this assumption does not hold, and a special treatment is needed—see Achdou et al. [1] for such analysis. Nevertheless, in many impulse control problems such as the one we refer to in the literature cited above, this separability assumption is satisfied.

### Organization

In Sect. 2 we set up the equilibrium of the mean field game in the simplest case of a one-dimensional variable. We define the decision maker’s problem, the aggregation, how the equilibrium relates them as a fixed point, and introduce the concept of the Impulse Hamiltonian. In Sect. 3 we present three decision problems that give rise to an Impulse Hamiltonian. In Sect. 4 we develop the result of uniqueness of the equilibrium for the mean field game. In Sect. 5 we introduce an exogenous aggregate random variable, in addition to the idiosyncratic state controlled by the decision maker. In Sect. 6 we extend the set up to a multidimensional case and prove uniqueness. For this case we use a version with noisy control, but the extent of the noise can be taken to be arbitrarily small.

## Set up

We describe the elements that define a MFG. Let $\mathcal {P} \equiv \{ m:\mathbb {R} \rightarrow \mathbb {R}_+, \int m(x) dx =1\}$ be the space of densities, and $0<T<\infty$ be the time horizon. A MFG is defined by $\{ \rho , F, H, \mu , \sigma , m_0, u_T\}$ where: (i) $\rho \ge 0$ is the discount rate, (ii) $F:\mathbb {R}\times \mathcal {P} \rightarrow \mathbb {R}$ is the flow cost with global coupling, (iii) $H:\mathbb {R}_{+} \times \mathbb {R} \rightarrow \mathbb {R}_{-}$ is the Impulse-Hamiltonian, (iv) $\mu :\mathbb {R} \rightarrow \mathbb {R}$, and $\sigma :\mathbb {R} \rightarrow \mathbb {R}_+$ are the drift and diffusion coefficients of the uncontrolled state, (v) $m_0 \in \mathcal {P}$ is the initial cross sectional density, (vi) $u_T: \mathbb {R}\times \mathcal {P} \rightarrow \mathbb {R}$ is the terminal value function.

The equilibrium of the MFG is given by the triplet $\{ \bar{x},u,m\}$: (a) a path for the optimal reset point $\bar{x}:[0,T] \rightarrow \mathbb {R}$, (b) a value function $u:\mathbb {R} \times [0,T] \rightarrow \mathbb {R}$, and (c) a cross-sectional density $m:\mathbb {R} \times [0,T] \rightarrow \mathbb {R}_+$. This triplet has to solve the coupled Hamilton–Jacobi–Bellman equation, and the corresponding Kolmogorov Forward equation described next.

### Hamilton–Jacobi–Bellman equation

Given a path $\{ m(x,t)\}$, the value function *u* and the path $\bar{x}$ solves the following HJB equation and boundary conditions:

1 $$\begin{aligned} \rho u(x,t)&= H\left( u(x,t)-u(\bar{x}(t) , t) , x \right) + F(x,m(t)) + \mathcal {L}\left( u(x,t) \right) \nonumber \\&\quad + \partial _t u(x,t)\quad \text {for all}\quad t\in [0,T], x \in \mathbb {R} \end{aligned}$$

2 $$\begin{aligned} u(x,t)&\ge u(\bar{x}(t),t) \quad \text {for all}\quad t\in [0,T], x \in \mathbb {R} \end{aligned}$$

3 $$\begin{aligned} u(x,T)&= u_T(x,m(T))\quad \text {for all}\quad x \in \mathbb {R} \end{aligned}$$

where $\mathcal {L}$ gives the expected change of the value function per unit of time due to the change in *x*, and for any function $f(\cdot ,t)$ that is twice differentiable is defined as

$$\begin{aligned} \mathcal {L}\left( f \right) (x,t) = \mu (x) \partial _x f(x,t) +\tfrac{1}{2} \sigma ^2(x) \partial _{xx} f(x,t) \end{aligned}$$

Equation (2) defines $\bar{x}(t)$ as the optimal reset point at *t*, i.e., it defines the optimal decision for an agent that can adjust her state. Finally, (3) specifies that when the game ends at $t=T$, the decision maker receives the given terminal reward $u_T$.

### Interpretation of the Impulse-Hamiltonian

The Impulse-Hamiltonian $H: \mathbb {R}_+ \times \mathbb {R }\rightarrow \mathbb {R}_{-}$ gives the expected change (decrease) in the value function per unit of time when the highest difference between the current value function and the minimum of the value function is $\nu$, i.e., $\nu = u(x,t)-u(\bar{x}(t),t)$, conditional on the current state *x*. This change occurs as the consequence of an optimal impulse, i.e., a discrete adjustment, that changes the state from *x* to $\bar{x}(t)$, i.e., from the current value *x* to the value that minimizes $u(\cdot ,t)$. We let $\bar{\lambda }(\nu ,x)$ be the probability (per unit of time) of an adjustment given $\nu$. We impose that $H(0,x) = 0$ for all *x*, since at the optimum there is nothing that can be gained by an adjustment. We postulate that $\bar{\lambda }$ is positive, and increasing in $\nu$, i.e.,

$$\begin{aligned} \bar{\lambda }(\nu ,x) \ge 0, \bar{\lambda }(0,x) = 0 ,\quad \text {and}\quad \bar{\lambda }_{\nu }(\nu ,x) > 0. \end{aligned}$$

We postulate that $H_\nu$ is (minus) the probability of adjustment per unit of time, i.e.,

$$\begin{aligned} H_\nu (\nu ,x) = - \bar{\lambda }(\nu ,x) \quad \text {and}\quad H_{\nu \nu }(\nu ,x) = - \bar{\lambda }_\nu (\nu ,x) < 0. \end{aligned}$$

Two comments are in order. Firstly as of now it is not clear why (minus) the derivative of the Impulse Hamiltonian equals the probability (per unit of time) of adjustment. In Sect. 3 we present three problems where we *derive* the Impulse Hamiltonian featuring the properties assumed here. Secondly we note that the first derivative of the Impulse Hamiltonian gives what Caballero and Engel [9] and Alvarez et al. [5] call the *generalized hazard function*

$$\begin{aligned} \Lambda (x,t) \equiv \bar{\lambda }( u(x,t) - u(\bar{x}(t),t) ) = - H_\nu (u(x,t) - u(\bar{x}(t),t),x). \end{aligned}$$

### Fokker–Planck–Kolmogorov forward equation

Given the value function $\{u(x,t) \}$ and path $\{\bar{x}(t)\}$ the cross sectional density $m \ge 0$ solves the partial differential equation

4 $$\begin{aligned} \partial _t m(x,t) = \mathcal {L}^*\left( m \right) (x,t) - \bar{\lambda }\left( u(x,t) - u(\bar{x}(t),x) , x\right) \, m(x,t) \end{aligned}$$

for all $t\in [0,T]$, all $x \in \mathbb {R}, x\ne \bar{x}(t)$, where $\mathcal {L}^*$ is defined at *x* for a function $f(\cdot ,t)$ that is twice differentiable as

$$\begin{aligned} \mathcal {L}^*\left( f \right) (x,t) = - \partial _x \left( \mu (x) f(x,t)\right) +\tfrac{1}{2} \partial _{xx} \left( \sigma ^2(x) f(x,t)\right) . \end{aligned}$$

We remark that the term $\bar{\lambda }\left( u(x,t)- u(\bar{x}(t),x), x\right) \, m(x,t)$ is the probability flux that leaves the state (*x*, *t*) as a consequence of the optimal adjustment. Moreover, this flux is the product of the probability of adjustment $\bar{\lambda }\left( u(x,t)- u(\bar{x}(t),x), x\right)$ per unit of time, multiplied by the density at that point *m*(*x*, *t*). Finally, (4) does * not * apply at $(x,t) = (\bar{x}(t), t)$. In this point there is an influx coming in from all the other points (*x*, *t*) with $x\ne \bar{x}(t)$, so its evolution is not local. We return to this issue in Lemma 1. Alternatively we could have written (4), for all *x* and $t \in [0,T]$, as:

5 $$\begin{aligned} \partial _t m(x,t) = \mathcal {L}^*\left( m \right) (x,t) - \bar{\lambda }\left( u(x,t) - u(\bar{x}(t),x) , x\right) \, m(x,t) + \delta (x-\bar{x}(t)) E(t) \end{aligned}$$

where $E(t) \equiv \int \bar{\lambda }(x,t) m(x,t) dx$, and where the derivatives in this equation should be interpreted in the sense of distributions. Notice that (5) includes the term with a delta function localized at $\bar{x}(t)$ times the flow of exit per period *E*(*t*). The extra term $\delta (x-\bar{x}(t)) E(t)$ has the interpretation of the flow of entry into $x=\bar{x}(t)$ per period.^5^

We can rewrite the time evolution of *m* using the assumed property of the Impulse Hamiltonian, namely that $\bar{\lambda }(\nu ,x) = -H_\nu \left( \nu , x\right)$ is the probability of an adjustment per unit of time if $\nu = u(x,t) -u(\bar{x}(t),t)$. Using this property, we can rewrite the time evolution, for all $t\in [0,T]$, $x \in \mathbb {R}, x\ne \bar{x}(t)$, as

6 $$\begin{aligned} \partial _t m(x,t) = \mathcal {L}^*\left( m \right) (x,t) + H_\nu \left( u(x,t)- u(\bar{x}(t),x), x\right) \, m(x,t). \end{aligned}$$

We can also write a version of (6) that holds for all *x*, using the delta function and including the term $E(t) \delta (x-\bar{x}(t))$ as was done in (5).

Finally, to completely determine the time evolution, we require that *m* must preserve the probability and that it is initialized by $m_0$, namely for all $x \in \mathbb {R}$ and for all $t \in [0,T]$,

7 $$\begin{aligned} 1&= \int _{-\infty }^\infty m(x,t) dx ,\end{aligned}$$

8 $$\begin{aligned} m_0(x)&= m(x,0) , \end{aligned}$$

where we note that the density *m* cannot be differentiable at $\bar{x}$ due to the incoming flow of agents that are re-injected there after adjustment, as further discussed below.

### Relative value function

Note that the argument of *H* in the Hamilton–Bellman–Jacobi equation and of $H_\nu$ in the Kolmogorov forward equation is $u(x,t) -u(\bar{x}(t),t)$. Motivated by this, we define the function *v*, for all *x* and for all $t \in [0,T]$, as

9 $$\begin{aligned} v(x,t) = u(x,t) -u(\bar{x}(t),t) \end{aligned}$$

We are now ready to define a classical equilibrium for the MFG.

#### Definition 1

Fix an initial density $m_0$ and a terminal value $u_T$. A classical equilibrium of the MFG is a triplet of functions $\{u, m, \bar{x}\}$ where (a) $\{u, \bar{x}\}$ satisfies the p.d.e. (1), the boundary conditions in (2), and the terminal condition in (3) given *m*, and where (b) *m* satisfies the p.d.e. (6), the condition in (7), and the initial condition in (8) given $\{u, \bar{x}\}$.

## Three examples of impulse Hamiltonian

We present here three decision problems where we can define the impulse Hamiltonian with the properties introduced above. Wang [22] and Dupuis and Wang [17] also study similar decision problems in one dimension, which can be written using an Impulse Hamiltonian. Menaldi and Robin [20] study a decision problem in many dimensions, a more general setup that cannot be accommodated by the Impulse Hamiltonian.

In Appendix B we use a discrete-time discrete-state model to explain the foundations of these problems. We first define the control problem of the decision maker in sequence form, and the corresponding Bellman equation. The discrete time notation is chosen so that the corresponding continuous time formulation is immediate. Finally we use the discrete time Bellman equation to formally derive the continuous time HJB equation for each model, namely (10) and (14) used in the body of the paper.

### Costly probabilistic adjustment

In this example, the decision maker pays a flow cost $c(\lambda ,x)$ and obtains a probability per unit of time of changing the state, $\lambda$, i.e., she decides the Poisson arrival rate of an opportunity to change the state. The cost depends on the current level of the state *x* and on the chosen probability $\lambda$. If the opportunity materializes, which occurs with probability $\lambda$, the decision maker can choose the level of the state freely, and she will do so to minimize $u(\cdot , t)$. The model here is a generalization of the one in Woodford [23] and Costain and Nakov [16].

The value function *u*(*x*, *t*) solves (see Appendix B.1)

10 $$\begin{aligned} \rho u(x,t)&= u_t\left( x,t\right) +\mathcal {L}\left( u \right) (x,t)+ F(x,m(t)) \ \end{aligned}$$

11 $$\begin{aligned}&\quad + \min _{\lambda \ge 0}\left[ c(\lambda ,x) - \lambda \left( u(x,t) - \min _y u(y,t) \right) \right] \quad \text {for all}\quad x \in \mathbb {R}\quad \text {and}\quad t \in [0,T] \nonumber \\&\quad \text {and} \nonumber \\ u(x,T)&= u_T(x,m(T))\quad \text {for all}\quad x \in \mathbb {R}, \end{aligned}$$

where the path of *m*(*t*) is taken as given by the decision maker.

We assume that the cost function *c* depends on the probability $\lambda$ and the current value of the state *x*, i.e., $c:\mathbb {R}_{+}\times \mathbb {R} \rightarrow \mathbb {R}$, and that, for each $x \in \mathbb {R}$, $c(\cdot ,x)$ is differentiable with

$$\begin{aligned} c(\lambda ,x)&\ge 0, \quad c(0,x) = 0, \quad c_\lambda (\lambda ,x) \ge 0,\quad c_\lambda (0,x) = 0, \quad \text {and}\\ c_{\lambda \lambda }(\lambda ,x)&>0\quad \text {if}\quad c_\lambda (\lambda ,x) >0. \end{aligned}$$

We now rewrite this problem introducing $\bar{x}(t) = \arg \min _y u(y,t)$, i.e., the optimal choice $\bar{x}(t)$, for all $t \in [0,T]$. Then we define the impulse Hamiltonian for any $\nu \ge 0$ as

12 $$\begin{aligned}&H(\nu ,x) = \min _{\lambda \ge 0} c(\lambda ,x) - \lambda \nu \end{aligned}$$

13 $$\begin{aligned}&\text {and the optimal choice}\ \bar{\lambda }(\nu ,x) = \arg \min _{\lambda \ge 0} c(\lambda ,x) - \lambda \nu , \end{aligned}$$

with the following properties:

1. $$H(0,x)=0$$.
2. $$H(\nu ,x) \le 0$$, and $$H_\nu (\nu ,x) < 0$$ for $$\nu >0$$.
3. $$H_\nu (\nu ,x) = - \bar{\lambda }(\nu ,x) $$ solving $$ \nu = c_\lambda \left( \bar{\lambda }(\nu ,x),x\right) $$ for $$\nu >0$$.
4. $$H_{\nu \nu }(\nu ,x) =- \bar{\lambda }_\nu (\nu ,x) = -1/c_{\lambda \lambda }\left( \bar{\lambda }(\nu ,x),x\right) < 0$$ for $$\nu >0$$.

For future reference note that, if there is an $\underline{\ell }>0$ such that $c(\lambda ,x)=0$ for all $\lambda \in [0,\underline{\ell }]$ and *x*, then

$$\begin{aligned} H_\nu (\nu ,x) \le - \underline{\ell }<0. \end{aligned}$$

Also, if there is $\underline{L}>0$ such that $c_{\lambda \lambda } (\lambda ,x)>\underline{L}>0$ for all $\lambda > \underline{ l}$ and *x*, then

$$\begin{aligned} H_{\nu \nu }(\nu ,x) \le -\underline{L}<0\quad \text {for all}\quad \nu ,x. \end{aligned}$$

### Random fixed costs

In this case, with probability $\kappa (x) >0$ per unit of time, the decision maker draws a fixed cost of adjustment $\psi$ from a distribution with a mass point $G(0,x)\ge 0$ for $\psi =0$, and with a density $g(\psi ,x)$ for $\psi >0$. The mass point *G* and density *g* are allowed to depend on the state *x*, so $g:(0,\infty ) \times \mathbb {R} \rightarrow [0,\infty )$. It is assumed that successive draws of the fixed cost, conditional on *x*, are independently distributed. A decision maker with state (*x*, *t*) and a realization $\psi$ of the cost at hand can either pay the cost $\psi$ and adjust, changing its value function from *u*(*x*, *t*) to $\psi +u(\bar{x}(t),x)$ where $\bar{x}(t) = \arg \min _x u(x,t)$, or not pay the fixed cost and let the state *x* evolve as uncontrolled. This is a continuous time version of the decision problem in Caballero and Engel [11–14], as characterized in Alvarez et al. [5].

The value function solves (see Appendix B.2)

14 $$\begin{aligned} \rho u(x,t)&= u_t\left( x,t\right) + \mathcal {L}\left( u\right) (x,t)+ F(x,m(t)) + \kappa (x) G(0,x) \left( u(\bar{x}(t),t) -u(x,t) \right) \nonumber \\&\quad + \kappa (x) \int _0^\infty \min \left\{ 0 \, , \, \psi \, + u(\bar{x}(t),t) - u(x,t) \right\} g(\psi ,x) d\psi \end{aligned}$$

15 $$\begin{aligned}&\quad \text {for all}\quad x \in \mathbb {R}\quad \text {and}\quad t \in [0,T] \nonumber \\&\text {and}\nonumber \\ u(x,T)&= u_T(x,m(T))\quad \text {for all}\quad x \in \mathbb {R}, \end{aligned}$$

where the path of *m*(*t*) is taken as given by the decision maker.

For this case we define, for any $\nu \ge 0$, the function $H: \mathbb {R}_+\times \mathbb {R} \rightarrow \mathbb {R}$. In this model,

16 $$\begin{aligned} H(\nu ,x)&= \kappa (x)\left[ -G(0,x) \nu + \int _0^\infty \min \left\{ 0 \, , \, \psi \,- \nu \right\} g(\psi ,x) d\psi \right] \nonumber \\&= \kappa (x)\left[ -G(0,x)\nu + \int _0^{\nu }\left( \psi - \nu \right) g(\psi ,x) d\psi \right] , \end{aligned}$$

17 $$\begin{aligned} \text { and observe that } \nonumber \\ H_\nu (\nu ,x)&= - \kappa (x) \left[ G(0,x) + \int _0^{\nu } g(\psi ,x) d\psi \right] \le 0\quad \text {so that} \nonumber \\ \bar{\lambda }(\nu ,x)&= \kappa (x) \left[ G(0,x)+\int _0^{\nu } g(\psi ,x) d\psi \right] \ge 0,\quad \text {and} \end{aligned}$$

18 $$\begin{aligned} H_{\nu \nu }(\nu ,x)&= - \kappa (x) g(\nu ,x) \le 0 \text { so that } \bar{\lambda }_\nu (\nu ,x) = \kappa (x) g(\nu ,x) \ge 0 . \end{aligned}$$

For future reference we note that, if there is an $\underline{\ell }>0$ such that $\kappa (x) G(0,x)\ge \underline{\ell }>0$ for all *x*, then

$$\begin{aligned} H_\nu (\nu ,x) \le -\underline{\ell }<0. \end{aligned}$$

Moreover, if there is $\underline{L}>0$ such that $\kappa (x) g(\nu ,x) \ge \underline{L}$, then

$$\begin{aligned} H_{\nu \nu }(\nu ,x) \le - \underline{L}<0. \end{aligned}$$

### Asymmetric random fixed costs

We present here a variation of the problem in Sect. 3.2. Given that the model is closely related to the problem in that section, we give fewer details. As in the previous case, when the Poisson clock with intensity $\kappa$ gives the agent an opportunity to adjust, she draws a *pair* of fixed cost $\psi ^+,\psi ^-$ from a CDF *G*. If the agent decides to increase its value of *x*, i.e., if $x(t^+)=\bar{x}(t)> x$, then the agent pays $\psi ^+$. If the agent decides to decrease the value of *x*, i.e., if $x(t^+)=\bar{x}(t)< x$, then the agent pays $\psi ^-$. If the agent does not change *x*, then she does not pay any cost. This specification allows a very asymmetric adjustment of increases and decreases, by specifying different marginal distributions of $\psi ^+$ and $\psi ^-$. One example is to make adjustment that implies decreases prohibitively costly, so if an adjustment occurs it is an increase. The reader can verify that this specification implies an impulse Hamiltonian with the same properties discussed in Sect. 3.2. A capital investment model allowing for asymmetric adjustment costs is presented and mapped to the data in Baley and Blanco [6] and Lippi and Oskolkov [19].

## Uniqueness of MFG: benchmark case

We prove the uniqueness of the classical one-dimensional MFG. We start by stating the assumptions that we use on the flow cost function *F*, the terminal value function $u_T$, the drift and volatility $\mu ,\sigma ^2$, and the impulse Hamiltonian *H*. Then we present two lemmas, the second one being the key for the proof, and state the main result. We need the following assumptions.

1. *Monotonicity assumption*. *F* and $u_T$ are *weakly monotone* if, for any $m^a, m^b \in \mathcal {P}$,
  19 $$\begin{aligned}&\int _{-\infty }^{\infty } \left( F(x,m^a)- F(x,m^b) \right) \left( m^a(x) - m^b(x)\right) dx \ge 0, \end{aligned}$$
  20 $$\begin{aligned}&\quad \text {and} \nonumber \\&\int _{-\infty }^{\infty } \left( u_T(x,m^a)- u_T(x,m^b) \right) \left( m^a(x) - m^b(x)\right) dx \ge 0, \end{aligned}$$
  *F* or $u_T$ are *strictly monotone* if the inequalities hold strictly every time that $\int _{-\infty }^{\infty } (m^a -m^b)^2 dx >0$.
2. *Boundedness of* *F* *and* $u_T$. There exists $B>0$ s.t., for all $m \in \mathcal {P} \text { and } x \in \mathbb {R}, | F(x,m) | \le B$ and $|u_T(x,m) | \le B$.
3. *Regularity of drift and volatility.* The drift $\mu (\cdot )$ is once continuously differentiable, $\sigma (\cdot )$ is twice continuously differentiable, and for some $B>0$
  21 $$\begin{aligned}&|| \sigma ||_{\infty } \le B, \, ||\mu ||_{\infty }\le B, \, ||\partial _x\mu ||_{\infty } \le B, \, ||\partial _x\sigma ||_\infty \le B \end{aligned}$$
4. *Impulse Hamiltonian*. The impulse Hamiltonian is nonpositive and twice continuously differentiable and, for all *x* and $\nu >0$,
  22 $$\begin{aligned} H(0,x)=0, \quad H_\nu (\nu ,x)\le 0 \quad \text {and}\quad H_{\nu \nu }(\nu ,x) \le 0. \end{aligned}$$

To state the uniqueness result it is necessary to revisit the definition of the MFG to add some regularity conditions.

### Definition 2

Let $\{ u, m, \bar{x}\}$ be a classical equilibrium of the MFG, specified as in Definition 1. We say that $\{ u, m, \bar{x}\}$ is a classical regular equilibrium if in addition:

1. $\bar{x}$ is a continuous function of time,
2. *u* is once continuously differentiable with respect to *t*, twice continuously differentiable with respect to *x* for all *t* with
  23 $$\begin{aligned} \partial _x u(x,t) \rightarrow 0 \quad \text {as}\quad |x| \rightarrow 0 \end{aligned}$$
3. *m* is continuous on (*x*, *t*) and, for all $t \in [0,T]$, $\int _{-\infty }^\infty m(x,t) dx =1$ (mass preservation), *m* is once continuously differentiable with respect to *t* everwhere, and twice continuously differentiable with respect to *x* for $(-\infty ,\bar{x}(t))$ and $(\bar{x}(t), \infty )$ with
  24 $$\begin{aligned} \partial _x m(x,t) \rightarrow 0 \quad \text {as}\quad |x| \rightarrow \infty , \end{aligned}$$

The next lemma uses the conservation of probability to obtain a useful equality between the adjustment flows, a property implied by the mass conservation of the problem.

### Lemma 1

Assume that *m* solves the Kolmogorov forward equation given $\{v, \bar{x}\}$ as described by (6), (7) and (8). Assume that *m* satisfies the integrability conditions of a classical regular equilibrium in (24), and that $\sigma ^2,\mu$ satisfies the assumptions in (21). Then, for all $t \in [0,T]$, we have

25 $$\begin{aligned} \tfrac{1}{2}\sigma ^2(\bar{x}(t))\left[ \partial _x m(\bar{x}(t)_{-},t) - \partial _x m(\bar{x}(t)_{+},t) \right] = - \int _{-\infty }^{\infty } m(x,t) H_\nu ( v(x,t),x) dx. \end{aligned}$$

For each *t* the left hand side of (25) is the difference between the probability inflow and outflow at $\bar{x}(t)$. Note that this requires that $\partial _x m(\cdot ,t)$ has a different right and left limit.

Given two classical regular equilibria of the MFG $\{u^a,m^a,\bar{x}^a,\}$ and $\{u^b,m^b, \bar{x}^b\}$ we define for all $t\in [0,T]$

26 $$\begin{aligned} K(t) \equiv \int _{-\infty }^{\infty } \left( u^a(x,t)- u^b(x,t)\right) \left( m^a(x,t)-m^b(x,t)\right) dx. \end{aligned}$$

The next Proposition obtains the key result to establish uniqueness, in a similar manner to the classical Lasry–Lions inequality.

### Proposition 1

Assume that *F* and $u_T$ are both bounded, and that they satisfies the weak monotonicity conditions given by (19), and that *H* is weakly decreasing and weakly concave as given by (22). Furthermore, assume that $\mu$ and $\sigma$ are once and twice continuously differentiable in *x*. Suppose that $\{u^a,m^a,\bar{x}^a\}$ and $\{u^a,m^b, \bar{x}^b\}$ are two classical regular equilibria of the MFG and let $v^a, v^b$ be defined as in (9). Then for all $t \in [0,T]$

27 $$\begin{aligned} \frac{d}{dt} K(t)&\le \rho K(t) \nonumber \\&\quad + \int _{-\infty }^{\infty } m^a(x,t) \Big [ \Big ( v^a(x,t)-v^b(x,t)\Big ) H_\nu (v^a(x,t),x) \nonumber \\&\quad + H(v^b(x,t),x) - H(v^a(x,t),x)\Big ] dx \nonumber \\&\quad + \int _{-\infty }^{\infty } m^b(x,t) \Big [\Big ( v^b(x,t)-v^a(x,t)\Big )H_\nu (v^b(x,t),x)\nonumber \\&\quad + H(v^a(x,t),x) - H(v^b(x,t),x)\Big ] dx \nonumber \\&\quad - \int _{-\infty }^{\infty } \left( F\left( x,m^a(t) \right) - F\left( x,m^b(t) \right) \right) \left( m^a(x,t) - m^b(x,t) \right) dx \end{aligned}$$

Moreover, $K(t)=0$ for all $t \in [0,T]$.

The strategy of the proof of Proposition 1 is similar to the one pioneered by Lasry and Lions. There are three differences. Firstly, $m(\cdot , t)$ is not differentiable at $x=\bar{x}(t)$. This brings an extra complication, which can be dealt in one dimension by careful analysis of the terms at $x=\bar{x}^a$ and $x=\bar{x}^b$. Indeed, as the reader can check in the proof, as a result of this lack of differentiability the right hand side of (27) contains an extra term which is bounded by $\sup _s H_\nu (s,x)[ v^a(\bar{x}^b(t),t) + v^b(\bar{x}^a(t),t)]\le 0$. Lemma 1 is used to control this term. Secondly, the p.d.e.’s for the HJB uses the Impulse Hamiltonian *H* which depends on $v(x,t) = u(x,t)-u(\bar{x}(t),t)$, as opposed to the classical Hamiltonian which depends on the first space derivative of *u* (i.e., derivative with respect to *x*) in the drift control case. Thirdly the p.d.e. for the KFE is coupled using the level of the Impulse Hamiltonian *H*, as opposed to the divergence in the case of the drift control case. Nevertheless, the convexity properties of the Impulse Hamiltonian allow a similar proof strategy as the classical Lasry and Lions result. Finally, the integrability conditions assumed in Definition 2 are used here for *K*(*t*) and its time derivative. Before comparing with the drift control, note that (27), can also be written, under the simplifying assumption that $\bar{H}_{\nu \nu }$ is a uniform upper bound on the second derivative $H_{\nu \nu }(\nu ,x)$, as follows:

28 $$\begin{aligned} \frac{d}{dot} K(t)&\le \rho K(t) + \frac{\bar{H}_{\nu \nu }}{2} \int _{-\infty }^{\infty } \left[ m^a(x,t) + m^b(x,t) \right] \left( v^a(x,t)- v^b(x,t)\right) ^2 dx \nonumber \\&\quad - \int _{-\infty }^{\infty } \left( F\left( x,m^a(t) \right) - F\left( x,m^b(t) \right) \right) \left( m^a(x,t) - m^b(x,t) \right) dx \end{aligned}$$

To be concrete, we recall that in the drift control setting $\tilde{u}^i, \tilde{m}^i$ are the value functions and distributions, and to simplify let $\tilde{\bar{H}}_{pp}<0$ be a uniform upper bound on the second derivative of the corresponding classical Hamiltonian $\tilde{H}(p,x)$, then the Lasry–Lions inequality, for

$$\begin{aligned} \tilde{K}(t)&\equiv \int _{-\infty }^{\infty } \left( \tilde{u}^a(x,t) - \tilde{u}^b(x,t) \right) \left( \tilde{m}^a(x,t) - \tilde{m}^b(x,t) \right) dx \quad \text {and}\\ \frac{d}{dt} \tilde{K}(t)&= \rho \tilde{K}(t) + \frac{\tilde{\bar{H}}_{pp}}{2} \int _{-\infty }^{\infty } \left( \tilde{m}^a(x,\tau )+ \tilde{m}^b(x,\tau )\right) \left( \partial _x \tilde{u}^a(x,\tau )- \partial _x \tilde{u}^b(x,\tau )\right) ^2 dx \\&\quad -\int _{-\infty }^{\infty } \left( F\left( x,\tilde{m}^a(t) \right) - F\left( x,\tilde{m}^b(t) \right) \right) \left( \tilde{m}^a(x,t) - \tilde{m}^b(x,t) \right) dx \end{aligned}$$

This expression is to be compared with (28) for our case.

Using the previous two lemmas we can show the main result, i.e., the uniqueness of the classical MFG equilibrium.

### Theorem 1

Assume that (i) *F* and $u_T$ are bounded, (ii) *F* is strictly monotone and $u_T$ weakly monotone as defined in (19) and (20), (iii) *H* satisfies the conditions given by (22) with $H_{\nu \nu }(v,x)<0$ for all $v>0$, and that (iv) $\mu$ and $\sigma$ are once and twice continuously differentiable in *x*. Let $\{u^a, m^a, \bar{x}^a \}$ and $\{u^b, m^b, \bar{x}^b \}$ be two classical regular MFG equilibrium as in Definition 2 for the initial distribution $m_0$ and terminal value $u_T$. Then $m^a=m^b$, $u^a=u^b$, and $\bar{x}^a = \bar{x}^b$, i.e., a classical regular equilibrium is unique.

The previous theorem is the main result of this paper. We remark that our proof uses both strict concavity of *H*, i.e., $H_{\nu \nu }<0$, and strict LL monotonicity of *F*. The strict monotonicity of *F* is what is required for uniqueness of Nash in the static non-atomic games. Nevertheless, in the standard classical MFG of drift control, sufficient conditions for uniqueness are weak monotonicity of *F* and weak convexity of $\tilde{H}$, with either of them holding in the strict sense. We believe that in our case, at the cost of a more cumbersome proof, uniqueness will follow from both weak concavity of $H_{\nu \nu }$ and weak LL monotonicity of *F*, and with either one of the two holding in the strict sense. Finally, the theorem states that the triplet $\{ u,m,\bar{x}\}$ is unique. Nevertheless, the uniqueness of the triplet does not imply that $u(\cdot , t)$ has a unique minimum.

The boundedness condition for *F* and $u_T$ can be exchanged by other integrability conditions, which implies that integrals of *v* are well defined, the limits of *v*(*x*, *t*) are bounded as |*x*| diverges. Likewise conditions (23) and (24) can be relaxed and still control the boundaries in several of the terms in the integration by parts.

## Adding an exogenous aggregate state

We consider here the case where the state of the problem is given by a triplet (*x*, *z*, *t*), where *x* is the state that can be controlled and affects the flow cost, while *z* is the state, common to all agents, that cannot be controlled, and both affect the flow cost. In this problem, the decision maker controls the probability of an adjustment of *x*, given the state (*x*, *z*, *t*). If an adjustment takes place, then the state will go from (*x*, *z*) at time *t* to $(\bar{x}(z,t),z)$ so $\bar{x}(z,t)$ is the optimally chosen value of *x*. The value function *u* has arguments (*x*, *z*, *t*). The Impulse Hamiltonian depends on the decrease in cost, conditional on adjustment, which we denote by $u(x,z,t)-u(\bar{x}(z,t),z,t)$ as well as on (*x*, *z*). Two economic examples giving rise to problems with such a structure will be presented.

The common state *z* follows a diffusion $dz=\hat{\mu }(z) dt + \hat{\sigma }(z) d\hat{W}$, where $\hat{W}$ is a standard Brownian motion. In this case the law of motion for the density of *z* can be written as:

29 $$\begin{aligned} \partial _t n(z,t) = - \partial _z \left( \hat{\mu }(z) n(z,t) \right) + \partial _{zz} \left( \frac{\hat{\sigma }^2(z)}{2} n(z,t) \right) . \end{aligned}$$

To simplify the analysis we assume that the cross sectional distribution of *z* is at steady state, and hence we omit the *t* index and simply write

$$\begin{aligned} 0 = - \partial _z \left( \hat{\mu }(z) n(z) \right) + \partial _{zz} \left( \frac{\hat{\sigma }^2(z)}{2} n(z) \right) . \end{aligned}$$

### The Hamilton–Jacobi–Bellman equation

Given a path $\{ m(x,z,t)\}$, the value function *u* and the path $\bar{x}(z,t)$ solves the following HJB equation and boundary conditions, for all $t\in [0,T]$ and $x \in \mathbb {R}$

30 $$\begin{aligned} \rho u(x,z,t)&= H\left( u(x,z,t)-u(\bar{x}(z,t),z,t), x,z \right) \nonumber \\&\quad + F(x,z,m(t)) + \mathcal {L}\left( u \right) (x,z,t) + \partial _t u(x,z,t), \end{aligned}$$

31 $$\begin{aligned} u(x,z,t)&\ge u(\bar{x}(t,z),z,t), \end{aligned}$$

32 $$\begin{aligned} u(x,z,T)&= u_T(x,z,m(T)), \end{aligned}$$

where $\mathcal {L}$ gives the expected change of the value function per unit of time, due to the change in *x*, *z*, and is defined for a twice continuously differentiable function *f*, as

$$\begin{aligned} \mathcal {L}\left( f \right) (x,z,t)&= \hat{\mu }(z) \partial _z f(x,z,t) + \mu (x,z) \partial _x f(x,z,t) \\&\quad +\tfrac{1}{2} \sigma ^2(x,z) \partial _{xx} f(x,z,t) +\tfrac{1}{2} \hat{\sigma }^2(z) \partial _{zz} f(x,z,t). \end{aligned}$$

Note that the optimal reset $\bar{x}(z,t)$ depends both on *t* and *z*. So, upon an adjustment at time *t*, the state of the decision maker jumps from (*x*, *z*) to $(\bar{x}(z,t),z)$. Also, implicit in the notation for $\mathcal {L}$ is the simplifying hypothesis that the brownians for *x* and *z* are independent, i.e., at times where there is no adjustment we assume that $dx=\mu (x,z)dt + \sigma (x,z) dW$ where $\mathbb {E}[d\hat{W}, dW]=0$.

In this section, we define *v* as

33 $$\begin{aligned} v(x,z,t) = u(x,z,t) - u(\bar{x}(x,z),z,t). \end{aligned}$$

### Fokker–Planck–Kolmogorov Forward equation

Given the value function $\{u(x,z,t) \}$ and path $\{\bar{x}(z,t)\}$, the cross sectional density *m* solves, for all $t\in [0,T], \ (x,z) \in \mathbb {R}^2, x \ne \bar{x}(z,t)$, the partial differential equation

34 $$\begin{aligned} \partial _t m(x,z,t)&= \mathcal {L}^*\left( m(x,z,t) \right) +H_\nu \left( u(x,z,t)- u(\bar{x}(z,t),x) , x,z\right) \, m(x,t)\quad \text {and} \end{aligned}$$

35 $$\begin{aligned} 1&= \int _{-\infty }^\infty \int _{-\infty }^\infty m(x,z,t) dx dz, \end{aligned}$$

with initial condition $m(x,z,0) = m_0(x,z)$, and where $\mathcal {L}^*$ is defined at *x*, *z*, for a function $g(\cdot ,t)$ that is twice differentiable, as

$$\begin{aligned} \mathcal {L}^*\left( g \right) (x,z,t)&= - \partial _x \left( \mu (x,z) g(x,z,t)\right) +\tfrac{1}{2} \partial _{xx} \left( \sigma ^2(x,z) g(x,z,t)\right) \\&\quad - \partial _z \left( \hat{\mu }(z) g(x,z,t)\right) +\tfrac{1}{2} \partial _{zz} \left( \hat{\sigma }^2(z) g(x,z,t)\right) . \end{aligned}$$

As before, $-H_\nu \left( u(x,z,t) - u(\bar{x}(z,t),x), x,z\right)$, gives the optimally chosen probability of adjustment.

The initial condition for *m* has to satisfy

36 $$\begin{aligned} m(x,z,0) = m_0(x,z)\quad \text {for all}\quad x,z\quad \text {with}\quad \int _{-\infty }^{\infty } m_0(x,z) dx = n(z)\quad \text {for all}\quad z. \end{aligned}$$

We define the conditional probability as:

37 $$\begin{aligned} m(x,t|z) = m(x,z,t) / n(z,t) = m(x,z,t) / n(z) \end{aligned}$$

and note that, for each (*z*, *t*),

38 $$\begin{aligned} \int _{-\infty }^{\infty } m(x,z,t) dx = n(z)\quad \text {and}\quad \int _{-\infty }^{\infty } \partial _t m(x,z,t) dx =0. \end{aligned}$$

### Two “monotone” price setting examples

We provide two price setting examples that give rise to the setup described above. In both examples *x* denotes the price charged by the firm, and the firm can only change its price if it pays a cost, which can be a costly probability of adjustment as in Sect. 3.1, or a random menu cost as in Sect. 3.2. In the first example the marginal cost of the firm depends on the output of the rest of the industry. In the second example the demand of the firm depends on the average price charged by the other firms. Both cases are simple enough so that checking monotonicity is easily done.

**Example 1: Input price dependent on industry output.** The demand is given by *D*(*x*, *z*), which we assume to be decreasing in the price *x*, and *z* is an exogenous shock. The marginal cost of the firm depends on the price of the input, which in turn is a function of the industry’s output. The industry output is given by $\bar{D} \equiv \int D(x',z') m(x',z') dx' dz'$, where *m* is the cross sectional distribution of firms indexed by *x*, *z*. The marginal cost is given by $c(\bar{D},z) = \gamma (z) + \theta \bar{D},$ where $\theta$ is a constant. Since we will be minimizing the objective function, we let *F* be minus the profits. Then the flow cost, i.e., “minus the profits”, is

$$\begin{aligned} F(x,z,m)&= - \left[ x - c\left( \bar{D},z\right) \right] D(x,z) = - \left[ x - \gamma (z) - \theta \bar{D} \right] D(x,z) \equiv \tilde{F} (x,z,\bar{D}). \end{aligned}$$

To discuss whether the static game defined by $\tilde{F}$ features strategic complementarity or substitutability, we introduce $x^*$, the optimal price chosen by the firm in the static game, given by

$$\begin{aligned} x^*( \bar{D})=\arg \min _x \tilde{F}(x,z,\bar{D}). \end{aligned}$$

A straightforward computation gives

$$\begin{aligned} \frac{ \partial x^*( \bar{D})}{\partial \bar{D}}= - \theta \frac{D_x(x^*,z,\bar{D})}{ \tilde{F}_{xx}(x^*,z,\bar{D})}. \end{aligned}$$

Note that $\bar{D}$ is smaller if *m* is replaced by $m'$ that is stochastically higher, i.e., if all firms charge higher prices the demand is lower. Hence $\frac{ \partial x^*( \bar{D})}{\partial \bar{D}}<0$ corresponds to the case of strategic complements in the static game, and $\frac{ \partial x^*( \bar{D})}{\partial \bar{D}}>0$ corresponds to the case of strategic substitutes. Hence, since $\tilde{F}_{xx}(x^*,z,\bar{D})\ge 0$ because $x^*$ is a minimum and since $D_x(x^*,z,\bar{D})<0$ because demand is decreasing in the price, the case where $\theta >0$ is one where the firms’ prices are strategic substitutes in the static game.

We can easily check weak monotonicity in this example. Indeed, let $m^a(x,z) = f^a(x|z) n(z)$ and $m^b(x,z) = f^b(x|z) n(z)$. Then (omitting the extremes of integration for simplicity)

$$\begin{aligned}&\int \int \left( F(x,z,m^a)- F(x,z,m^b)\right) (dm^a - dm^b) \\&\quad = \int n(z) \int \left( F(x,z,m^a)- F(x,z,m^b)\right) (f^a - f^b) dx dz\\&\quad = \theta \left[ \int n(z) \left( \int D(x,z) f^a(x|z) dx - \int D(x,z) f^b(x|z) dx \right) dz\right] ^2 \ge 0 \iff \theta >0. \end{aligned}$$

Thus monotonicity requires that the marginal cost increases with the industry output, which in this problem implies that the firms’ prices are strategic substitutes in the static game.

**Example 2: Cross demand elasticity dependent on the average price.** The demand for the good of the firm depends on its own price *x* and on the average price $X \equiv \int x' \, dm(x',z')$. We consider the following demand $D(x,z,X) = D_0(x,z) - \theta X$, where $\theta$ is a constant. Note that $\theta <0$ corresponds to the case where the goods are substitutes in the demand system, while $\theta >0$ corresponds to the case where the goods are complements in the demand system. We assume that the marginal cost is given by $\gamma (z)$. Then the flow cost, i.e., “minus the profits”, is (omitting the extremes of integration)

$$\begin{aligned} F(x,z,m)&= - \left( x - \gamma (z)\right) \left( D_0(x,z) - \theta X\right) \\&= -(x-\gamma (z)) D_0(x,z) - \gamma (z) \theta \int \int x' m(x',z') dx' dz'\\&\quad + \theta x \int \int x' m(x',z') dx' dz'\\&\equiv \tilde{F}(x,z,X). \end{aligned}$$

Notice that substitution or complementarity in the demand system are different than “strategic” substitution or complementarity used in game theory as referred to the agent’s actions. We now turn to discuss the strategic complementarity or substitution in the static game by introducing

$$\begin{aligned} x^*(X)=\arg \min _x \tilde{F}(x,z,X). \end{aligned}$$

A straightforward computation gives

$$\begin{aligned} \frac{ \partial x^*(X)}{\partial X}= - \theta \frac{1}{ \tilde{F}_{xx}(x^*,z,X)}, \end{aligned}$$

where we notice that $\tilde{F}_{xx}(x^*,z,X)\ge 0$ because $x^*$ is a minimum, so that $\frac{ \partial x^*(X)}{\partial X}<0$ implies that the prices are strategic substitutes in the static game.

We can easily check monotonicity in this example. Let $X^k \equiv \int \int x \, m^k(x,z) dx dz$ for $k=a,b$, so that (omitting the extremes of integration)

$$\begin{aligned}&\int \left( F(x,z,m^a)- F(x,z,m^b)\right) (dm^a - dm^b) \\&\quad = \theta \left[ X^a \int \int x \, (m^a(x,z) -m^b(x,z)) dxdz - X^b \int \int x \, (m^a(x,z)-m^b(x,z)) \, dxdz \right] \\&\quad = \theta (X^a-X^b)^2 \ge 0 \iff \theta >0. \end{aligned}$$

Thus monotonicity requires that the prices are strategic substitutes in the static game.

### Uniqueness of equilibrium of the MFG

We first list the assumptions on $\mu , \hat{\mu }, \sigma ^2, \hat{\sigma }^2$, *F* and $u_T$, and then give the regularity assumptions on the equilibrium objects $u,m,\bar{x}$. For the exogenous objects we assume that

1. Boundedness of $\mu$ and $\sigma$ and their *x*, *z* derivatives are bounded.
2. Boundedness of $\hat{\mu }$ and $\hat{\sigma }$ and their *z* derivatives are bounded.
3. The functions *F* and $u_T$ are continuous and bounded.
4. The impulse Hamiltonian *H*(*v*, *x*, *z*) is twice continuously differentiable in *v*, with $H_\nu (v, x,z)\le 0$ and $H_{\nu \nu }(v, x,z)\le 0$.

The definition of monotonicity is the same, i.e., we say that $f:\mathbb {R}^2 \times \mathcal {P} \rightarrow \mathbb {R}$ is weakly monotone if, for any two densities $m^a,m^b$

$$\begin{aligned} \int _{-\infty }^\infty \int _{-\infty }^\infty \left( f(x,z,m^a) - f(x,z,m^b) \right) \left( m^a(x,z) - m^b(x,z)\right) dx dz \ge 0 \ . \end{aligned}$$

In the definition of a classical regular equilibrium we require $u,m,\bar{x}$ to be smooth and integrable as follows

1. The function $\bar{x}(z,t)$ is a continuous function of time.
2. The function *u* is once continuously differentiable on *t* and twice continuously differentiable on (*x*, *z*) derivatives for each *t*.
3. The density *m* is integrable, its *x* derivatives converge to zero as $|x| \rightarrow \infty$ for each *t*. Likewise, its *z* derivatives converge to zero as $|z| \rightarrow \infty$.

Now we start with the characterization. The next lemma is a generalization of the analysis of the probability flux for the benchmark case.

#### Lemma 2

For any $z \in \mathbb {R}$ and $t \in (0,T)$, if *m* solves the KFE, we have

39 $$\begin{aligned}&\tfrac{1}{2}\sigma ^2(\bar{x}(z,t),z)\left[ \partial _x m(\bar{x}_{-}(z,t),z,t) - \partial _x m(\bar{x}_{+}(z,t),z,t) \right] \nonumber \\&\quad = - \int _{-\infty }^{\infty } m(x,z,t) H_\nu ( v(x,z,t),x,z) \, dx . \end{aligned}$$

For future reference we define

$$\begin{aligned} v(x,z,t) \equiv u(x,z,t) - u(\bar{x}(z,t),z),t) \text { for all } x,z,t \end{aligned}$$

as the natural extension of our previous definition for *v* in the benchmark case. Likewise, we define *K*(*t*, *z*) and $\bar{K}(t)$ as

40 $$\begin{aligned} K(t,z)&\equiv \int _{-\infty }^\infty \left( u^a(x,z,t) - u^b(x,z,t) \right) \left( m^a(x,z,t) - m^b(x,z,t)\right) dx \quad \text {and} \nonumber \\ \bar{K}(t)&\equiv \int _{-\infty }^\infty K(t,z) dz \nonumber \\ \bar{K}(t)&= \int _{-\infty }^{\infty } \int _{-\infty }^{\infty }\left( u^a(x,z,t)-u^b(x,z,t)\right) \left( m^a(x,z,t)-m^b(x,z,t)\right) dx dz \end{aligned}$$

By following an appropriately adapted version of the steps used in the benchmark case, we can show the main step for the uniqueness of equilibrium, i.e., a version of the Lasry–Lions inequality. This is done in the next proposition.

#### Proposition 2

Let $\{u^a,\bar{x}^a,m^a\}$ and $\{u^b,\bar{x}^b,m^b\}$ be two classical regular equilibria of the MFG, and assume that (i) *F* and $u_T$ are bounded, (ii) that *F* is LL weakly monotone, and that (iii) *H* is decreasing and concave. Let $\bar{K}$ be defined as (40). Then

$$\begin{aligned} \frac{d}{dt} \bar{K}(t)&\le \rho \bar{K}(t) - \int _{-\infty }^\infty \int _{-\infty }^\infty \left( f(x,z,m^a(t)) - f(x,z,m^b(t)) \right) \\ {}&\quad \left( m^a(x,z,t) - m^b(x,z,t)\right) dx dz \\&\quad + \int _{-\infty }^{\infty } \int _{-\infty }^{\infty } m^a(x,z,t) \left( v^a(x,z,t) - v^b(x,z,t)\right) H_\nu (v^a(x,z,t),x,z) dx dz \\&\quad + \int _{-\infty }^{\infty } \int _{-\infty }^{\infty } m^b(x,z,t) \left( v^b(x,z,t) - v^a(x,z,t)\right) H_\nu (v^b(x,z,t),x,z) dx dz \\&\quad + \int _{-\infty }^{\infty } \int _{-\infty }^{\infty } \left( m^a(x,z,t) - m^b(x,z,t) \right) \\&\quad \left[ H(v^b(x,a,t),x,z) - H(v^a(x,z,t),x,z)\right] dx dz \end{aligned}$$

The uniqueness of equilibrium can be obtained following similar steps as in the benchmark case. As in the benchmark case, it does require a stricter version of monotonicity for $f(\cdot )$, and a stricter concavity of *H*, i.e., that $H_{\nu \nu }<0$. Nevertheless, the key step for the uniqueness result is the inequality in Proposition 2. We leave the details of the uniqueness result to the reader, but they are completely parallel to the ones of the benchmark case.

## Multidimensional, noisy optimal decision

In this section we develop a version of the model where we use two generalizations. Firstly, we allow for $x\in \mathbb {R}^n$ for $n\ge 1$, i.e., we consider the multidimensional case. Secondly, when the decision maker decides to adjust the state, instead of jumping to $\bar{x}(t)$, the adjustment is distributed with a density $\eta _\epsilon$ centered around $\bar{x}(t)$. This distribution is indexed by $\epsilon >0$, where $\epsilon$ measures the dispersion of *x* around $\bar{x}(t)$. The distribution $\eta _\epsilon$ is given by

$$\begin{aligned} \eta _\epsilon (x-\bar{x}(t)) = \epsilon g\left( \frac{x-\bar{x}(t) }{\epsilon }\right) \end{aligned}$$

where $g:\mathbb {R} \rightarrow \mathbb {R}_+$ is a smooth density with a maximum at zero, so $g(0)> g'(0)=0$. We use $\eta _\epsilon$ to smooth out the choice of optimal reset point, i.e., when the decision maker selects to move the state from *x* to $\bar{x}(t)$, the state will be randomly distributed around $\bar{x}(t)$ according to the density $\eta _\epsilon$.

In this case the impulse Hamiltonian is $H:\mathbb {R}_+\times \mathbb {R}^n \rightarrow \mathbb {R}_{-}$. The function $H(\cdot ,x)$, for a fixed $x\in \mathbb {R}^n$, has the same properties as before, i.e., it is continuously differentiable, negative, decreasing, and concave. Moreover its first argument is the (negative) of the expected change conditional on an adjustment.

We note that we let $x\in \mathbb {R}^n$, the decision maker’s decision to exercise control changes the entire $n-$dimensional state, i.e., it changes all the components of the state. This is similar to the multiproduct pricing model of Midrigan [21], Alvarez and Lippi [4], and Bhattarai and Schoenle [8]. An interesting alternative assumption, which we explore separately in Sect. 5, is the one in which the decision maker can only control some subset of the state.

Additionally, each of the *n* coordinates of *x*, when it is uncontrolled, follows $dx_i = \mu _i(x) dt + \sigma _i(x) dW_i$ for $i=1,2,\ldots ,n$, where to simplify the notation we assume that $\{ W_i,W_j\}$ are orthogonal for $i\ne j$, i.e., $\mathbb {E}\left[ dW_i \, dW_j \right] =\delta _{i,j} \, dt$, where $\delta _{i,j}$ is the Kronecker delta. Thus we have two vectors fields

$$\begin{aligned} \mu (x) = \Big ( \mu _1(x),\mu _2(x) , \ldots , \mu _n(x)\Big )\quad \text {and}\quad \sigma ^2(x) = \Big ( \sigma ^2_1(x),\sigma ^2_2(x) , \ldots , \sigma ^2_n(x)\Big ). \end{aligned}$$

Corresponding to this process, or to $\mu$ and $\sigma ^2$, we have that for any smooth function $f:\mathbb {R}^n\times [0,T]\rightarrow \mathbb {R}$, we define the operators $\mathcal {L}(f)$ and $\mathcal {L}^*(f)$ by

$$\begin{aligned} \mathcal {L}(f)(x,t)&\equiv \sum _{i=1}^n \mu _i(x) \frac{\partial }{\partial x_i} f(x,t) + \frac{1}{2} \sum _{i=1}^n \sigma ^2_i(x) \frac{\partial ^2}{\partial x_i \partial x_i} f(x,t), \\&\quad \text {and} \\ \mathcal {L}^*(f)(x,t)&\equiv -\sum _{i=1}^n \frac{\partial }{\partial x_i} \left( f(x,t) \mu _i(x) \right) + \frac{1}{2} \sum _{i=1}^n \frac{\partial ^2 }{\partial x_i \partial x_i}\left( f(x,t) \sigma ^2_i(x)\right) . \end{aligned}$$

We also adapt the notation for the set of densities $\mathcal {P} = \big \{ f:\mathbb {R}^n\rightarrow \mathbb {R}_+ \text { with } \int f(x) dx =1 \big \}$. Likewise we adapt the conditions on the vector fields for the drift and volatility. In particular, the function $\mu :\mathbb {R}^n \rightarrow \mathbb {R}^n$ is once continuously differentiable, and $\sigma :\mathbb {R}^n \rightarrow \mathbb {R}^n_+$ is twice continuously differentiable, and there exists $B>0$ such that, for all $x\in \mathbb R^n$ and all $j=1,\ldots ,n$

$$\begin{aligned} || \sigma ||_{\infty } \le B,\quad ||\mu ||_{\infty }\le B, \quad ||\partial _x\mu ||_{\infty }\le B , \quad ||\partial _x\sigma ||_\infty \le B \end{aligned}$$

Next we define a classical regular equilibrium, for a given scalar $\epsilon$ as follows:

### Definition 3

Fix $\epsilon >0$ and let $(u_T,m_0)$ be given. A classical regular $\epsilon$-MFG is a triplet $(u,\bar{x},m)$, where $u:\mathbb {R}^n\times [0,T] \rightarrow \mathbb {R}$ and $m:\mathbb {R}^n\times [0,T] \rightarrow \mathbb {R}_+$ are once continuously differentiable with respect to *t*, and twice continuously differentiable with respect to *x*, and $\bar{x}: [0,T] \rightarrow \mathbb {R}^n$ is continuous on *t*, such that:

- $$||\partial _{x} u(x,t) ||_2 \rightarrow 0 \text { and } || \partial _x m(x,t)||_2 \rightarrow 0 \text { as } || x || \rightarrow \infty $$
- The HBJ in $(x,t) \in \mathbb {R}^n\times (0,T)$ is
  41 $$\begin{aligned} \rho u(x,t)&= F(x,m(t)) + \mathcal {L}(u)(x,t) + u_t(x,t) \nonumber \\&\quad + H\left( u(x,t) - \int _{\mathbb {R}^n} u(z,t) \eta _\epsilon (z-\bar{x}(t)) dz, x \right) \end{aligned}$$
- The KFE in $(x,t) \in \mathbb {R}^n\times [0,T]$ is
  42 $$\begin{aligned} m_t(x,t)&= H_\nu \left( u(x,t) - \int _{\mathbb {R}^n} u(z,t)\eta _\epsilon (z-\bar{x}(t))dz, x \right) m(x,t) + \mathcal {L}^*(m)(x,t) \nonumber \\&\quad -\left[ \int _{\mathbb {R}^n} H_\nu \left( u(x',t) - \int _{\mathbb {R}^n} u(z,t) \eta _\epsilon (z-\bar{x}(t))dz, x' \right) m(x',t) dx'\right] \nonumber \\&\quad \eta _\epsilon (x-\bar{x}(t)). \end{aligned}$$
- The optimal reset, for all $t \in [0,T]$, satisfies $\bar{x}(t) \in \arg \min _{x} \int u(z,t) \, \nu _{\epsilon }(z-x) \, dz.$
- The terminal and initial conditions for all $x\in \mathbb {R}^n$ are:
  43 $$\begin{aligned} u(x,T)&= u_T(x,m(T)) \text { and } m(x,0) = m_0(x). \end{aligned}$$

A few comments on the Definition 3 are in order:

1. It is convenient to adapt the definition of *v* to the case of noisy control to read as follows
  44 $$\begin{aligned} v(x,t) = u(x,t) - \int _{\mathbb {R}^n} u(z,t) \eta _\epsilon (z-\bar{x}(t)) dz. \end{aligned}$$
2. As in the benchmark case, the term $-H_\nu \left( v(x,t), x \right) \ge 0$ gives the probability of an adjustment at (*x*, *t*).
3. The term $-\left[ \int _{\mathbb {R}^n} H_\nu \left( v(x',t),x'\right) m(x',t) dx'\right] \eta _\epsilon (x-\bar{x}(t))$ in the KFE is the product of the fraction of the values which adjust, given by $-\int _{\mathbb {R}^n} H_\nu (v(x',t),x') m(x') dx'$, times the density of those that adjust to the value *x*, given by $\eta _\epsilon (x-\bar{x}(t))$. This is the probability flow that “enters" at the value *x* at time *t*.
4. The KFE holds for all *x*, including $x=\bar{x}(t)$. This differs from the benchmark case with $\epsilon =0$. This difference, i.e., the fact that *m* is smooth everywhere, is due to the regularizing effect of the function $\eta _\epsilon$.
5. As $\epsilon \downarrow 0$, then $\eta _\epsilon$ becomes a delta function, and then the KFE holds with a Dirac mass at $(x,t)=(\bar{x}(t),t)$.
6. If $\epsilon =0$ and $n=1$ we have exactly the baseline case. If $\epsilon =0$ and $n>1$ we would have the extension of the baseline case to the case where the decision maker controls the entire $n-$dimensional state, a case which we don’t treat directly.
7. As in the baseline case, $\mathcal {L}$ gives the ($n-$dimensional version of the) linear operator describing the effect on the expected change on *u* of the drift $\mu (\cdot )$ and volatility $\sigma (\cdot )$.
8. As in the baseline case, $\mathcal {L}^*$ gives the ($n-$dimensional version of the) linear operator describing the propagation of the density *m* due to effect of the drift $\mu (\cdot )$ and volatility $\sigma (\cdot )$.

We define weak monotonicity in the same way:

45 $$\begin{aligned}&\int _{\mathbb {R}^n} \left( F(x,m^a)- F(x,m^b) \right) \left( m^a(x) - m^b(x)\right) dx \ge 0 \quad \text {for all}\quad m^a, m^b \in \mathcal {P} , \end{aligned}$$

46 $$\begin{aligned}&\int _{\mathbb {R}^n} \left( u_T(x,m^a)- u_T(x,m^b) \right) \left( m^a(x) - m^b(x)\right) dx \ge 0 \quad \text {for all}\quad m^a, m^b \in \mathcal {P}, \end{aligned}$$

where $$\mathcal {P} = \left\{ f:\mathbb {R}^n\rightarrow \mathbb {R}_+ \text { with } \int _{\mathbb {R}^n} f(x) dx =1 \right\} $$.

The next proposition is the key to show that a classical $\epsilon$-MFG equilibrium is unique under the analogous assumptions as in the baseline case. It establishes a Lasry–Lions type of inequality, which is the key result to show the uniqueness of equilibrium. In particular we have:

### Proposition 3

Assume that $\epsilon >0$, that *F* and $u_T$ are bounded, that *F* satisfies the monotonicity conditions given by (45), and that *H*(*v*, *x*) is decreasing and concave in *v*, and twice continuously differentiable. Furthermore, assume that $\mu$ and $\sigma$ are bounded and once and twice continuously differentiable in $x\in \mathbb {R}^n$. Suppose that $\{u^a,m^a,\bar{x}^a\}$ and $\{u^b,m^b,\bar{x}^b\}$ are two classical equilibria of the $\epsilon$-MFG as stated in Definition 3. Let $v^a, v^b$ be defined as in (44), and

47 $$\begin{aligned}&K(t) \equiv \int _{\mathbb {R}^n} \Big ( u^a(x,t)- u^b(x,t)\Big ) \Big ( m^a(x,t)-m^b(x,t)\Big ) dx \text { for all } t \in [0,T]. \end{aligned}$$

48 $$\begin{aligned}&\text {Then}\,\,\frac{d}{dt} K(t) \le \rho K(t) -\int _{\mathbb {R}^n} \Big (F(x,m^a(t))- F(x,m^b(t)) \Big ) \Big ( m^a(x,t) - m^b(x,t)\Big ) dx \nonumber \\&\quad + \int _{-\infty }^{\infty } m^a(x,t) \Big [\Big ( v^a(x,t)-v^b(x,t)\Big ) H_\nu (v^a(x,t),x) \nonumber \\&\quad + H(v^b(x,t),x) - H(v^a(x,t),x)\Big ] dx \nonumber \\&\quad + \int _{-\infty }^{\infty } m^b(x,t) \Big [\Big ( v^b(x,t)-v^a(x,t)\Big )H_\nu (v^b(x,t),x)\nonumber \\&\quad + H(v^a(x,t),x) - H(v^b(x,t),x)\Big ] dx \end{aligned}$$

for all $$t\in (0,T)$$.

From a technical point of view the proof of Proposition 3 is similar to the one for the benchmark case in Proposition 1. The main difference is that in the case of $\epsilon >0$, the density *m* is differentiable everywhere, so we don’t need to develop a special argument to bound the terms involving the discontinuities in $m_x(\cdot ,t)$ at $x=\bar{x}(t)$, as we need to do in the case of $\epsilon =0$. In the one dimensional case analyzed in Proposition 1, these terms were easier to deal with, involving the product of left and right derivatives of $m_x(\cdot ,t)$ at $x=\bar{x}(t)$ with other functions that we can sign. Instead, in the $n-$dimensional case with $\epsilon =0$, the arguments would have been much more cumbersome.

Given Proposition 3, the proof of uniqueness of the classical regular equilibria of the $\epsilon$-MFG follows similar steps as in the baseline one dimensional case with $\epsilon =0$. In particular, if we add weak monotonicity of $u_T$, strengthen the assumption to strict monotonicity of *F* and to strict concavity of *H* with $H_{\nu \nu }<0$, then the analogous steps lead to the uniqueness of the equilibrium.

An interesting question are the properties of the limit as $\epsilon \downarrow 0$. Recall that we have shown that the $\epsilon$-MFG equilibrium is unique for $\epsilon >0$. Is the limit of an $\epsilon$-MFG as $\epsilon \downarrow 0$ always an equilibrium for the $n-$dimensional model with $\epsilon =0$? Is it the case that any equilibrium for $\epsilon =0$ corresponds to the limit as $\epsilon \downarrow 0$ for an $n-$dimensional $\epsilon$-MFG model? We leave these questions for future work.

## Concluding remarks

Lasry and Lions [18] established an important uniqueness result for a Mean-Field-Game problem in which the decision maker controls *the drift* of a diffusion process. An important feature of the Lasry–Lions result is that the uniqueness is ensured by a property of the flow cost function, the so called Lasry–Lions monotonicity condition. This property is a form of “strategic substitutability” when applied to a game defined by the period cost function.

Our paper presented a complementary uniqueness result for some problems where the control of the decision maker consists in choosing the stopping times for resetting the state, rather than in controlling its drift. In other words, we examine the uniqueness of the equilibrium in a MFG characterized by a form of “Impulse Control” instead of “drift control”. The type of problem we consider is common in economics and emerges in e.g., optimal investment or price-setting problems in the presence of fixed costs, pioneered by Caballero and Engel [10, 12]. We analyzed three variations on the problem. We identify suitable assumptions that, coupled with the Lasry–Lions monotonicity condition, ensure that the problem features a unique classical equilibrium.

## Proofs

### Proof of Lemma 1

Differentiating (7) with respect to time, and replacing using (6), we obtain:

$$\begin{aligned} 0&= \int _{-\infty }^\infty \partial _t m(x,t) dx \\&=- \int _{-\infty }^\infty \partial _x\left( \mu (x) m(x,t) \right) dx + \int _{-\infty }^\infty \tfrac{1}{2} \partial _{xx} \left( \sigma ^2(x) m(x,t) \right) dx\\&\quad + \int _{-\infty }^\infty H_\nu (v(x,t),x) m(x,t) dx \\&=- \mu (x) m(x,t) |^\infty _{-\infty } + \tfrac{1}{2} \partial _{x} \left( \sigma ^2(x) m(x,t) \right) |^\infty _{\bar{x}_{+}(t)} + \tfrac{1}{2} \partial _{x} \left( \sigma ^2(x) m(x,t) \right) |^{\bar{x}_{-}(t)}_{-\infty } \\&\quad + \int _{-\infty }^\infty H_\nu (v(x,t),x) m(x,t) dx \end{aligned}$$

Using that $\mu (x)$ is bounded and $m(x,t)\rightarrow 0$ as $|x|\rightarrow \infty$, and that $\sigma ^2$ is bounded, and that $m_x(x,t)\rightarrow 0$ as $|x| \rightarrow \infty$ then

$$\begin{aligned} 0&= \tfrac{1}{2} \partial _{x} \left( \sigma ^2(x) m(x,t) \right) |^{\bar{x}_{-}(t)}_{\bar{x}_{+}(t)} + \int _{-\infty }^\infty H_\nu (v(x,t),x) m(x,t) dx \end{aligned}$$

Since $m(\cdot , t), \sigma ^2(\cdot ,t)$ and $\partial _x\sigma ^2(\cdot ,t)$ are continuous at $x=\bar{x}(t)$ then

$$\begin{aligned} \partial _{x} \left( \sigma ^2(x) m(x,t) \right) |^{\bar{x}_{-}(t)}_{\bar{x}_{+}(t)}&= \sigma ^2(\bar{x}(t)) \partial _{x} \left( \partial _x m(x,t) \right) |^{\bar{x}_{-}(t)}_{\bar{x}_{+}(t)} \end{aligned}$$

obtaining the desired result. $\square$

### Proof of Proposition 1

First we note that because *F* is bounded, then *u* is also bounded. Then the following integral is well defiend:

$$\begin{aligned} K(t)&= \int _{-\infty }^{\infty } \left( u^a(x,t)-u^b(x,t)\right) \left( m^a(x,t)-m^b(x,t)\right) dx \end{aligned}$$

and since $v(x,t)-u(x,t)=-u(\bar{x}(t),t)$ does not depend on *x*, then

$$\begin{aligned} \partial _x u(x,t) = \partial _x v(x,t), \text { and } \partial _{xx} u(x,t) = \partial _{xx} v(x,t) \text { for all } x . \end{aligned}$$

Using that $\int _{-\infty }^{\infty } \left( m^a(x,t)-m^b(x,t)\right) dx=0$, then:

$$\begin{aligned} K(t)&=\int _{-\infty }^{\infty } \left( v^a(x,t)-v^b(x,t)\right) \left( m^a(x,t)-m^b(x,t)\right) dx \end{aligned}$$

We differentiate *K* with respect to time to obtain:

$$\begin{aligned} \frac{d}{dt} K(t)&= \frac{d}{dt}\int _{-\infty }^{\infty } \left( v^a(x,t)-v^b(x,t)\right) \left( m^a(x,t)-m^b(x,t)\right) dx \\&=\int _{-\infty }^{\infty } \left( v^a(x,t)-v^b(x,t)\right) \partial _t \left( m^a(x,t)-m^b(x,t)\right) dx\\&\quad +\int _{-\infty }^{\infty } \left( m^a(x,t)-m^b(x,t)\right) \partial _t \left( v^a(x,t)-v^b(x,t)\right) dx \end{aligned}$$

The strategy is to exchange the time derivative of the integral with the integral of the time derivative, to replace the resulting time derivatives with the expression of the KFE, and then applying integration by parts. Using the properties of $v^i(x,t)-u^i(x,t)=-u^i(\bar{x}^i(t),t)$ for $i=a,b$, and that $\int (m^a-m^b)dx$ and its time derivatives are zero:

$$\begin{aligned} \frac{d}{dt} K(t)&= \frac{d}{dt}\int _{-\infty }^{\infty } \left( u^a(x,t)-u^b(x,t)\right) \left( m^a(x,t)-m^b(x,t)\right) dx \\&=\int _{-\infty }^{\infty } \left( v^a(x,t)-v^b(x,t)\right) \partial _t \left( m^a(x,t)-m^b(x,t)\right) dx\\&\quad +\int _{-\infty }^{\infty } \left( m^a(x,t)-m^b(x,t)\right) \partial _t \left( u^a(x,t)-u^b(x,t)\right) dx \end{aligned}$$

Using the p.d.e for the HJB in (1), using that $\partial _x v=\partial _x u$ and $\partial _{xx} v=\partial _{xx} u$, and the p.d.e in (6) to replace in the previous integrals as follows:

$$\begin{aligned}&\frac{d}{dt}\int _{-\infty }^{\infty } \left( u^a(x,t)-u^b(x,t)\right) \left( m^a(x,t)-m^b(x,t)\right) dx \\&\quad =\int _{-\infty }^{\infty } \left( v^a-v^b\right) \left( \partial _{xx}(\tfrac{\sigma ^2}{2}m^a-\tfrac{\sigma ^2}{2}m^b)\right) - \left( m^a-m^b\right) \tfrac{\sigma ^2}{2} \left( \partial _{xx}(v^a-v^b) \right) dx\\&\qquad +\int _{-\infty }^{\infty } \left( v^a-v^b\right) \left( - \partial _{x}(\mu \, m^a-\mu \, m^b)\right) - \left( m^a-m^b\right) \mu \left( \partial _{x}(v^a-v^b) \right) dx\\&\qquad +\rho \int _{-\infty }^{\infty } \left( u^a-u^b\right) \left( m^a-m^b\right) dx\\&\qquad + \int _{-\infty }^{\infty } \left( v^a-v^b\right) \left( m^a H_\nu (v^a)- m^b H_\nu (v^b)\right) dx - \int _{-\infty }^{\infty } \left( m^a-m^b\right) \left( H(v^a)- H(v^b)\right) dx \\&\qquad - \int _{-\infty }^{\infty }\left( m^a-m^b\right) \left( F(x,m^a)- F(x,m^b)\right) dx \end{aligned}$$

where we omit the arguments (*x*, *t*) or *x* from the different functions to simplify the notation. Thus we write:

$$\begin{aligned} \frac{d}{dt} K(t)&= \frac{d}{dt}\int _{-\infty }^{\infty } \left( u^a-u^b\right) \left( m^a-m^b\right) dx =\rho \int _{-\infty }^{\infty } \left( u^a-u^b\right) \left( m^a-m^b\right) dx \\&\quad + I_{V}(t)+ I_{D}(t)+I_H(t) +I_F (t) \end{aligned}$$

where

$$\begin{aligned} I_{V}(t)&\equiv \int _{-\infty }^{\infty } \left( v^a-v^b\right) \left( \partial _{xx}(\tfrac{\sigma ^2}{2}m^a-\tfrac{\sigma ^2}{2}m^b)\right) - \left( m^a-m^b\right) \tfrac{\sigma ^2}{2} \left( \partial _{xx}(v^a-v^b) \right) dx\\ I_{D}(t)&\equiv \int _{-\infty }^{\infty } \left( v^a-v^b\right) \left( - \partial _{x}(\mu \, m^a-\mu \, m^b)\right) - \left( m^a-m^b\right) \mu \left( \partial _{x}(v^a-v^b) \right) dx\\ I_H(t)&\equiv \int _{-\infty }^{\infty } \left( v^a-v^b\right) \left( m^a H_\nu (v^a)- m^b H_\nu (v^b)\right) dx\\&\quad - \int _{-\infty }^{\infty } \left( m^a-m^b\right) \left( H(v^a)- H(v^b)\right) dx \\ I_F(t)&\equiv - \int _{-\infty }^{\infty }\left( m^a-m^b\right) \left( F(m^a) - F(m^b)\right) dx \end{aligned}$$

Note that the integrals in $$I_{V}(t), I_{D}(t)$$, and $$I_H(t)$$ are all well defined given the integrability assumptions of a classical regular equilibrium, as well as the integrability assumptions on $$\mu $$ and $$\sigma ^2$$. Next, we obtain an inequality for each term, i.e., for $$I_{V}(t),I_{D}(t), I_H(t) $$ and $$I_F(t)$$.

1. We will show that
  $$\begin{aligned} I_V(t) \le \left[ v^b(\bar{x}^a(t),t) + v^a(\bar{x}^b(t),t) \right] \bar{H}_\nu \end{aligned}$$
  where $\bar{H}_\nu = \sup _{\{s\ge 0, x\}} H_{\nu }(s)$. To simplify the notation we let $\hat{v} \equiv v^a-v^b$ and $\hat{m} \equiv m^a-m^b$. With this notation we have
  $$\begin{aligned} I_{V}(t)|_L^U&\equiv \int _{L}^{U}\left[ \left( v^a-v^b\right) \left( \partial _{xx}(\tfrac{\sigma ^2}{2}m^a-\tfrac{\sigma ^2}{2}m^b)\right) - \left( m^a-m^b\right) \tfrac{\sigma ^2}{2} \left( \partial _{xx}(v^a-v^b) \right) \right] dx \\&= \int _{L}^{U} \left[ \hat{v} \, \partial _{xx}\left( \tfrac{\sigma ^2}{2} \hat{m} \right) - \hat{m} \, \tfrac{\sigma ^2}{2}\partial _{xx} \hat{v} \right] dx \end{aligned}$$
  In an interval $x\in [L,U]$ where $\hat{m}_x$ is twice continuously differentiable we have:
  $$\begin{aligned} I_{V}(t)|_L^U&= \int _{L}^{U} \left[ \hat{v} \, \partial _{xx}\left( \tfrac{\sigma ^2}{2} \hat{m} \right) - \hat{m} \, \tfrac{\sigma ^2}{2}\partial _{xx} \hat{v} \right] dx \\&= \int _{L}^{U} \hat{v} \, \partial _{xx}\left( \tfrac{\sigma ^2}{2} \hat{m} \right) dx + \int _{L}^{U} \partial _{x} \left( \hat{m} \tfrac{\sigma ^2}{2} \right) \partial _{x} \hat{v} dx - \hat{m} \tfrac{\sigma ^2}{2} \, \partial _{x} \hat{v} | ^U_L \\&= \hat{v} \, \partial _{x} \left( \hat{m} \tfrac{\sigma ^2}{2}\right) | ^U_L - \hat{m} \tfrac{\sigma ^2}{2} \, \partial _{x} \hat{v} | ^U_L \end{aligned}$$
  where we integrate by parts each term. Assume, without loss of generality that $-\infty< \bar{x}^a(t)< \bar{x}^b(t) < \infty$, so we can write:
  $$\begin{aligned} I_{V}(t)|_{-\infty }^\infty&= I_{V}(t)|_{-\infty }^{\bar{x}^a} + I_{V}(t)|_{\bar{x}^a}^{\bar{x}^b}+ I_{V}(t)|_{\bar{x}^b}^{\infty } \end{aligned}$$
  We use that, given the assumption on integrability for a classical regular equilibrium, then $m^i \rightarrow 0$ and $|\partial _x m^i | \rightarrow 0$, that $\partial _x v^i \rightarrow 0$ as $|x| \rightarrow \infty$, and $v^i$ is bounded for $i=a,b$ to obtain:
  $$\begin{aligned} 0=&\lim _{|x| \rightarrow \infty } \hat{v} \, \partial _{x} \left( \hat{m} \tfrac{\sigma ^2}{2}\right) (x) - \lim _{|x| \rightarrow \infty } \hat{m} \tfrac{\sigma ^2}{2} \, \partial _{x} \hat{v} (x) \end{aligned}$$
  Thus we can write:
  $$\begin{aligned} I_{V}(t)|_{-\infty }^\infty&= I_{V}(t)|_{\bar{x}^a_{+}}^{\bar{x}^a_{-}} + I_{V}(t)|_{\bar{x}^b_{+}}^{\bar{x}^b_{-}} \end{aligned}$$
  Let’s concentrate on the first term, $I_{V}(t)|_{\bar{x}^a_{+}}^{\bar{x}^a_{-}}$. We use that $v^a(\bar{x}^a(t),t)=0$ and $\partial _x v^a(x,t)|_{x=x^a}=0$ to obtain:
  $$\begin{aligned} I_{V}(t)|_{\bar{x}^a_{+}}^{\bar{x}^a_{-}}&= \hat{v} \, \partial _{x} \left( \hat{m} \tfrac{\sigma ^2}{2}\right) |_{\bar{x}^a_{+}}^{\bar{x}^a_{-}} - \hat{m} \tfrac{\sigma ^2}{2} \, \partial _{x} \hat{v} |_{\bar{x}^a_{+}}^{\bar{x}^a_{-}} \\&=\hat{v} \, \partial _{x} \left( \hat{m} \tfrac{\sigma ^2}{2}\right) |_{\bar{x}^a_{+}}^{\bar{x}^a_{-}} \end{aligned}$$
  where the second line uses that $m^i$ and $\partial _x v^i$ are continuous on *x*, and hence $0=\hat{m} \tfrac{\sigma ^2}{2} \, \partial _{x} \hat{v} |_{\bar{x}^a_{+}}^{\bar{x}^a_{-}}$. Then
  $$\begin{aligned} I_{V}(t)|_{\bar{x}^a_{+}}^{\bar{x}^a_{-}}&=\hat{v} \, \partial _{x} \left( \hat{m} \tfrac{\sigma ^2}{2}\right) |_{\bar{x}^a_{+}}^{\bar{x}^a_{-}} = - v^b \, \tfrac{\sigma ^2}{2} \, \partial _{x} m^a |_{\bar{x}^a_{+}}^{\bar{x}^a_{-}} < 0 \end{aligned}$$
  where we use that $\sigma ^2$ is continuously differentiable, and that $\hat{m}=m^a-m^b$, and that $\partial _x m^b$ is continuous at $x=\bar{x}^a$. Using Lemma 1 we obtain:
  $$\begin{aligned} I_{V}(t)|_{\bar{x}^a_{+}}^{\bar{x}^a_{-}}&= v^b(\bar{x}^a(t),t) \int _{-\infty }^{\infty } m^a(x,t) H_\nu ( v^a(x,t),x) dx \le v^b(\bar{x}^a(t),t) \bar{H}_\nu \end{aligned}$$
  The argument for $I_{V}(t)|_{\bar{x}^b_{+}}^{\bar{x}^b_{-}}$ is identical, giving:
  $$\begin{aligned} I_{V}(t)|_{\bar{x}^b_{+}}^{\bar{x}^b_{-}}&= v^a(\bar{x}^b(t),t) \int _{-\infty }^{\infty } m^b(x,t) H_\nu ( v^b(x,t),x) dx \le v^a(\bar{x}^b(t),t) \bar{H}_\nu \end{aligned}$$
  Combining the two results we obtain the desired inequality.
2. We will show that $I_D(t) = 0$. This follows by using integration by parts, since $\hat{v}(\cdot ,t)$ is continuously differentiable. The boundaries at $x=\infty$ and $x=-\infty$, the terms vanish given the assumption on the tails. In particular:
  $$\begin{aligned} I_D(t)&= \int _{-\infty }^{\infty } \left( v^a-v^b\right) \left( - \partial _{x}(\mu \, m^a-\mu \, m^b)\right) - \left( m^a-m^b\right) \mu \left( \partial _{x}(v^a-v^b) \right) dx \\&=- \left( v^a-v^b\right) \left( \mu \, m^a-\mu \, m^b\right) |^{\infty }_{-\infty } \end{aligned}$$
  using that $v^i(\cdot ,t)$, and $\mu$ are bounded, and that $m^i\rightarrow 0$ as $|x|\rightarrow \infty$. Thus $I_D(t)=0$.
3. We will show that
  $$\begin{aligned} I_H(t)&=\int _{-\infty }^{\infty } m^a(x,t) \Big [ \Big ( v^a(x,t)-v^b(x,t)\Big ) H_\nu (v^a(x,t),x)\\&\quad + H(v^b(x,t),x) - H(v^a(x,t),x)\Big ] dx \\&\quad + \int _{-\infty }^{\infty } m^b(x,t) \Big [ \Big ( v^b(x,t)-v^a(x,t)\Big )H_\nu (v^b(x,t),x)\\&\quad + H(v^a(x,t),x) - H(v^b(x,t),x)\Big ] dx \end{aligned}$$
  We fix a *x*, *t*, omit them for clarity and write:
  $$\begin{aligned}&\left( v^a-v^b\right) \left( m^a H_\nu (v^a)- m^b H_\nu (v^b)\right) - \left( m^a-m^b\right) \left( H(v^a)- H(v^b)\right) \\&\quad = m^a \left[ \left( v^a-v^b\right) H_\nu (v^a) + H(v^b) - H(v^a)\right] \\&\qquad + m^b \left[ \left( v^b-v^a\right) H_\nu (v^b) + H(v^a) - H(v^b)\right] \end{aligned}$$
  Integrating across *x* we get the desired expression. That $I_H(t) \le 0$ follows directly by concavity of $H(\cdot ,x)$.
4. Finally, $I_F(t)\le 0$. This follows directly by the assumption (19) on weak monotonicity for *F*.

Combining the bounds obtained by $I_{V}(t)$, $I_{D}(t)$, $I_H(t)$ and $I_F (t)$ we get the desired result in (27).

To finish the proof we use the inequality in (27), together with the assumptions of weak monotonicity of *F*, weak monotonicity $u_T$, and concavity of $H(\cdot ,x)$ to show that $K(t)=0$ for all $t\in [0,T]$. Note that, since $m^a,m^b$ are equilibrium, then they both satisfy $m^a(x,0)=m^b(x,0) =m_0(0)$, and thus $K(0)=0$. Then, using this initial condition, we can integrate (27), to obtain that for all $t\in [0,T]$:

49 $$\begin{aligned} K(t)&\le \end{aligned}$$

50 $$\begin{aligned}&\int _0^t e^{-\rho (t-s)} \int _{-\infty }^{\infty } m^a(x,s) \Big [ \Big ( v^a(x,s)-v^b(x,s)\Big ) H_\nu (v^a(x,s),x)\nonumber \\&\quad + H(v^b(x,s),x) - H(v^a(x,s),x)\Big ] dx \, ds \nonumber \\&\quad + \int _0^t e^{-\rho (t-s)} \int _{-\infty }^{\infty } m^b(x,s) \Big [ \Big ( v^b(x,s)-v^a(x,s)\Big )H_\nu (v^b(x,s),x)\nonumber \\&\quad + H(v^a(x,s),x) -H(v^b(x,s),x)\Big ] dx\, ds\nonumber \\&\quad - \int _0^t e^{-\rho (t-s)} \int _{-\infty }^{\infty } \left( F(x,m^a(s))-F(x,m^b(s)) \right) \left( m^a(x,s)-m^b(x,s) \right) dx \, ds \nonumber \\&\le 0 \end{aligned}$$

where the inequalities follow from the weak monotonicity of *F*, and weak concavity of $H(\cdot , x)$. By definition of an equilibrium, $u^i(x,T) =u_T(x,m^i(T))$ for $i=a,b$, so $K(T) = \int (u_T(x,m^a(T))-u_T(x,m^b(T))) (m^a(x,T)-m^b(x,T)) dx \ge 0$, where the inequality comes from the assumption of weak monotonicity of $u_T$. Thus for $K(T)\ge 0$ and (49) to hold simultaneously it must be that $K(t)=0$ for almost all *t*. $\square$

### Proof of Theorem 1

We proceed in three steps. The proof uses the results of Proposition 1. First we show that *m* is unique. Second, we show that $\bar{x}$ is unique. Third we show that *v* is unique. Fourth, we show that *u* is unique.

As an intermediate step, note that under the stated assumptions Proposition 1 holds, and hence $K(t)=0$ for all *t*. Thus, $\frac{d}{dt}K(t)=K(t)=0$ so using (27) we get that each of the two terms of the right hand side is non-positive, so that

51 $$\begin{aligned} 0&= \int _{-\infty }^{\infty } m^a(x,t) \Big [\Big ( v^a(x,t)-v^b(x,t)\Big ) H_\nu (v^a(x,t),x)\nonumber \\&\quad + H(v^b(x,t),x) - H(v^a(x,t),x)\Big ] dx \nonumber \\&\quad + \int _{-\infty }^{\infty } m^b(x,t) \Big [\Big ( v^b(x,t)-v^a(x,t)\Big )H_\nu (v^b(x,t),x)\nonumber \\&\quad + H(v^a(x,t),x) - H(v^b(x,t),x)\Big ] dx \end{aligned}$$

52 $$\begin{aligned}&\quad \text {and} \nonumber \\ 0&= \int _{-\infty }^{\infty } \left[ F(x,m^a(t)) - F(x,m^b(t)) \right] \left( m^a(x,t) - m^b(x,t) \right) dx \end{aligned}$$

*Step 1: Uniqueness of* *m*. Using the assumption of strict monotonicity of *F*, we have that (52) implies that $m^a=m^b$.

*Step 2: Uniqueness of* $\bar{x}$. Assume, to obtain a contradiction, that $\bar{x}^a(t) \ne \bar{x}^b(t)$ for some *t*. Recall that the derivative $\partial _x m^a(x,t)$ has a jump discontinuity at $x=\bar{x}^a(t)$. On the other hand, since $\bar{x}^b(t) \ne \bar{x}^a(t)$, then in classical MFG equilibrium, $\partial _x m^b(x,t)$ is differentiable at $x=\bar{x}^a(t)$. This is a contradiction with $m^a=m^b$, and hence $\bar{x}^a(t) = \bar{x}^b(t)$.

*Step 3: Uniqueness of* *v*. Since $m^a=m^b=m$, we can write (51) as:

53 $$\begin{aligned} 0&= \int _{-\infty }^{\infty } m(x,t) \left[ \left( v^a(x,t)-v^b(x,t)\right) H_\nu (v^a(x,t),x) + H(v^b(x,t),x) - H(v^a(x,t),x)\right] dx \nonumber \\&\quad + \int _{-\infty }^{\infty } m(x,t) \left[ \left( v^b(x,t)-v^a(x,t)\right) H_\nu (v^b(x,t),x) + H(v^a(x,t),x) - H(v^b(x,t),x)\right] dx \nonumber \\&= \int _{-\infty }^{\infty } m(x,t) \left( v^a(x,t)-v^b(x,t)\right) \left( H_\nu (v^a(x,t),x) -H_\nu (v^b(x,t),x)\right) dx \nonumber \\&= \int _{-\infty }^{\infty } m(x,t) \left( v^b(x,t)-v^a(x,t)\right) \int _{v^a(x,t)}^{v^b(x,t)} H_{\nu \nu }(s,x) ds \, dx \end{aligned}$$

Since we assume that $H_{\nu \nu }(s,x)<0$ for all $s>0$, then whenever $m(x,t)>0$ we must have $v^a(x,t)=v^b(x,t)$.

*Step 4: Uniqueness of* *u*. We use that $v^a=v^a$ and that $m^a=m^b$ to establish that $u^a=u^b$. To do so, we compare the HBJ for $u^a$ with the one for $u^b$, establishing three properties. First, since $v^a(x,t)=v^b(x,t)$ for all *x*, *t* then, $\partial _x u^a(x,t) = \partial _x u^b(x,t)$ and $\partial _{xx} u^a(x,t) = \partial _{xx} u^b(x,t)$, and hence $\mathcal {L}(u^a) = \mathcal {L}(u^b)$. Second, since $m^a=m^b$, we have $F(x,m^a(t))= F(x,m^b(t))$. Third, since $v^a=v^b$, then $H(v^a(x,t),x)= H(v^b(x,t),x)$. Thus, subtracting the p.d.e. for the HBJ of $u^b$ from the one for $u^a$ we get the following o.d.e. for every *x*:

$$\begin{aligned} \rho (u^a(x,t) - u^b(x,t) ) = \partial _t u^a(x,t) - \partial _t u^b(x,t) \text { for } t\in [0,T] \end{aligned}$$

with terminal condition $u^a(x,T) - u^b(x,T) =u_T(x,m^a(T))-u_T(x,m^b(T))=0$ since $m^a(\cdot ,T)=m^b(\cdot ,T)$. The only solution of this o.d.e. is zero. Thus, $u^a(x,t) - u^b(x,t)=0$ for all *x*, *t*. $\square$

### Proof of Lemma 2

Since $\int _{-\infty }^{\infty } m(x,z,t)dx =n(z)$ which does not depend on time:

$$\begin{aligned} 0&=\int _{-\infty }^{\infty } \partial _t m(x,z,t)dx \\&=- \int _{-\infty }^{\infty } \partial _x( \mu (x,z,t) m(x,z,t) ) dx - \int _{-\infty }^{\infty } \partial _z( \hat{\mu }(z) n(z) m(x,t|z) )dx \\&\quad + \int _{-\infty }^{\infty } \partial _{xx}( \tfrac{1}{2} \sigma ^2(x,z,t) m(x,z,t) ) dx + \int _{-\infty }^{\infty } \partial _{zz}( \tfrac{1}{2} \hat{\sigma }^2(z,t) n(z) m(x,t|z) ) dx \\&\quad +\int _{-\infty }^{\infty } H_\nu (v(x,z,t),x,z) m(x,z,t) dx \\&=- \int _{-\infty }^{\infty } \partial _x( \mu (x,z,t) m(x,z,t) ) dx - \partial _z( \hat{\mu }(z) n(z) )\int _{-\infty }^{\infty } m(x,t|z)dx \\&\quad - \hat{\mu }(z) n(z) \int _{-\infty }^{\infty } \partial _z( m(x,t|z) )dx \\&\quad + \int _{-\infty }^{\infty } \partial _{xx}( \tfrac{1}{2} \sigma ^2(x,z,t) m(x,z,t) ) dx +\partial _{zz}( \tfrac{1}{2} \hat{\sigma }^2(z,t) n(z) ) \int _{-\infty }^{\infty } m(x,t|z) dx \\&\quad + 2 \partial _{z}( \tfrac{1}{2} \hat{\sigma }^2(z,t) n(z) ) \int _{-\infty }^{\infty } \partial _{z} m(x,t|z) dx + \tfrac{1}{2} \hat{\sigma }^2(z,t)n(z) \int _{-\infty }^{\infty } \partial _{zz} m(x,t|z) dx \\&\quad +\int _{-\infty }^{\infty } H_\nu (v(x,z,t),x,z) m(x,z,t) dx \end{aligned}$$

Using the p.d.e for *n*:

$$\begin{aligned} 0&=- \int _{-\infty }^{\infty } \partial _x( \mu (x,z,t) m(x,z,t) ) dx \\&\quad - \hat{\mu }(z) n(z) \int _{-\infty }^{\infty } \partial _z m(x,t|z) dx + \int _{-\infty }^{\infty } \partial _{xx}( \tfrac{1}{2} \sigma ^2(x,z,t) m(x,z,t) ) dx \\&\quad + 2 \partial _{z}( \tfrac{1}{2} \hat{\sigma }^2(z,t) n(z) ) \int _{-\infty }^{\infty } \partial _{z} m(x,t|z) dx + \tfrac{1}{2} \hat{\sigma }^2(z,t)n(z) \int _{-\infty }^{\infty } \partial _{zz} m(x,t|z) dx \\&\quad +\int _{-\infty }^{\infty } H_\nu (v(x,z,t),x,z) m(x,z,t) dx \end{aligned}$$

Exchanging the integrals with derivatives:

$$\begin{aligned} 0&=- \int _{-\infty }^{\infty } \partial _x( \mu (x,z,t) m(x,z,t) ) dx \\&\quad - \hat{\mu }(z) n(z) \partial _z \int _{-\infty }^{\infty } m(x,t|z) dx + \int _{-\infty }^{\infty } \partial _{xx}( \tfrac{1}{2} \sigma ^2(x,z,t) m(x,z,t) ) dx \\&\quad + 2 \partial _{z}( \tfrac{1}{2} \hat{\sigma }^2(z,t) n(z) ) \partial _{z} \int _{-\infty }^{\infty } m(x,t|z) dx \\&\quad + \tfrac{1}{2} \hat{\sigma }^2(z,t)n(z) \partial _{zz} \int _{-\infty }^{\infty } m(x,t|z) dx +\int _{-\infty }^{\infty } H_\nu (v(x,z,t),x,z) m(x,z,t) dx \end{aligned}$$

But since for all *z*:

$$\begin{aligned} 1= \int _{-\infty }^{\infty } m(x,t|z) dx \text { then } 0= \partial _z \int _{-\infty }^{\infty } m(x,t|z) dx =\partial _{zz} \int _{-\infty }^{\infty } m(x,t|z) dx \end{aligned}$$

then

$$\begin{aligned} 0&=- \int _{-\infty }^{\infty } \partial _x( \mu (x,z,t) m(x,z,t) ) dx + \int _{-\infty }^{\infty } \partial _{xx}( \tfrac{1}{2} \sigma ^2(x,z,t) m(x,z,t) ) dx \\&\quad +\int _{-\infty }^{\infty } H_\nu (v(x,z,t),x,z) m(x,z,t) dx \end{aligned}$$

Using the boundedness of $\mu$ and integrability of *m*:

$$\begin{aligned} \int _{-\infty }^\infty \partial _x( \mu (x,z,t) m(x,z,t) ) dx = \mu (x,z,t) m(x,z,t) |^{x=\infty }_{x=-\infty } =0 \end{aligned}$$

Finally, using the boundedness and integrability of *m* at infinity, we get:

$$\begin{aligned}&\int _{-\infty }^{\infty } \partial _{xx}( \tfrac{1}{2} \sigma ^2(x,z,t) m(x,z,t) ) dx =\partial _{x}( \tfrac{1}{2} \sigma ^2(x,z,t) m(x,z,t) ) |^{x=\infty }_{x=-\infty } \\&\quad +\partial _{x}( \tfrac{1}{2} \sigma ^2(x,z,t) m(x,z,t) ) |^{x=\bar{x}_{-}(z,t)}_{x=\bar{x}_{+}(z,t)} =\partial _{x}( \tfrac{1}{2} \sigma ^2(x,z,t) m(x,z,t) ) |^{x=\bar{x}_{-}(z,t)}_{x=\bar{x}_{+}(z,t)}\\&= \tfrac{1}{2} \sigma ^2(\bar{x}(z),z,t) \partial _{x}( m(x,z,t) ) |^{x=\bar{x}_{-}(z,t)}_{x=\bar{x}_{+}(z,t)} \end{aligned}$$

Replacing these expression we obtain the desired result. $\square$

We write a series of lemmas which prepare the results for Proposition 2.

### Lemma 3

Let $\{ u^a, \bar{x}^a, m^a\}$ and $\{ u^b, \bar{x}^b, m^b\}$ be two classical regular equilibria of the MFG, and recall $v^i(x,z,t) \equiv u^i(x,z,t) - u^i(\bar{x}^i(z,t),z),t)$ for $i=a,b$. Then, for any $z \in \mathbb {R}$ and $t \in (0,T)$,

$$\begin{aligned} K(t,z)&\equiv \int _{-\infty }^\infty \left( u^a(x,z,t) - u^b(x,z,t) \right) \left( m^a(x,z,t) - m^b(x,z,t)\right) dx\\&= \int _{-\infty }^\infty \left( v^a(x,z,t) - v^b(x,z,t)\right) \left( m^a(x,z,t) - m^b(x,z,t)\right) dx. \end{aligned}$$

### Proof of Lemma 3

Let $g(z,t) = u^a(\bar{x}^a(z,t),z),t) - u^b(\bar{x}^b(z,t),z),t)$ then $\int _{-\infty }^\infty (u^a - u^b)(m^a - m^b) dx = \int _{-\infty }^\infty (v^a - v^b)(m^a - m^b) dx + \int _{-\infty }^\infty g (m^a - m^b)dx$. But $\int _{-\infty }^\infty m^a(x,z,t) dx = \int _{-\infty }^\infty m^b(x,z,t) dx = n(z)$ so that $\int _{-\infty }^\infty g(z,t) (m^a(x,z,t) - m^b(x,z,t))dx= g(z,t) [n(z)-n(z)]=0$. $\square$

The next lemma, which uses the p.d.e.’s for the HBJ and KF equations, gives a decomposition of the change of *K* through time (the extremes of integration are omitted to simplify notation).

### Lemma 4

Define $\bar{K}(t) = \int K(t,z) dz$. Then, for all $t\in [0,T]$,

$$\begin{aligned} \frac{d}{dt}\bar{K}(t)&= \rho \bar{K}(t) \\&\quad + \int \int \left( (v^a(x,z,t)-v^b(x,z,t) \right) \mathcal {L}^*(m^a-m^b)(x,z,t) dx dz \\&\quad - \int \int \left( (m^a(x,z,t)-m^b(x,z,t)\right) \mathcal {L}(v^a-v^b)((x,z,t) dx dz \\&\quad - \int \int \left( m^a(x,z,t)-m^b(x,z,t) \right) \left( F(x,z,m^a(t))-F(x,z,m^b(t))\right) dx dz \\&\quad - \int \int \left( (m^a(x,z,t)-m^b(x,z,t) \right) (H(v^a(x,z,t),x,z )\\&\quad - H(v^b(x,z,t),x,z )) dx dz \\&\quad + \int \int \left( (v^a(x,z,t)-v^b(x,z,t)\right) \\&\qquad \left( H_\nu (v^a(x,z,t),x,z) m^a(x,z,t) - H_\nu (v^b(x,z,t),x,z) m^b(x,z,t)\right) dx dz. \end{aligned}$$

where we omit the range of the integrals, which runs from $-\infty$ to $+\infty$ to simplify the notation.

### Proof of Lemma 4

We omit the range of the integrals, which runs from $-\infty$ to $+\infty$ to simplify the notation. Start with

$$\begin{aligned} \frac{d}{dt}\bar{K}(t)&= \int \frac{d}{dt} K(t,z) dz = \int \int \frac{d}{dt} (u^a-u^b)(m^a-m^b) dx dz \\&= \int \int (m^a-m^b) \partial _t (u^a-u^b) dx dz +\int \int (u^a-u^b) \partial _t (m^a-m^b) dx dz \end{aligned}$$

Using p.d.e. obtained from the HJB equation:

$$\begin{aligned}&\int \int (m^a-m^b) \partial _t (u^a-u^b) dx dz \\&\quad =\int \int (m^a-m^b) \rho (u^a-u^b)dx dz - \int \int (m^a-m^b) (F(m^a)-F(m^b)) dx dz\\&\qquad - \int \int (m^a-m^b) (H(v^a)-H(v^b)) dx dz - \int \int (m^a-m^b) \mathcal {L}(u^a-u^b) dx dz \end{aligned}$$

and using the p.d.e. obtained from the KF equation:

$$\begin{aligned}&\int \int (u^a-u^b) \partial _t (m^a-m^b) dx dz \\&\quad =\int \int (u^a-u^b) \mathcal {L}^*(m^a-m^b)dx dz + \int \int (u^a-u^b) (H_\nu (v^a) m^a-H_\nu (v^b)m^b) dx dz \end{aligned}$$

Use that $[u^a(x,z,t) - u^b(x,z,t)] - [ v^a(x,z,t)- v^b(x,z,t)] = g(z,t)$ where the function *g* does not depend on *x*, then

$$\begin{aligned}&\int \int (u^a-u^b) \partial _t (m^a-m^b) dx dz - \int \int (v^a-u^v) \partial _t (m^a-m^b) dx dz \\&\quad = \int \int g \partial _t (m^a-m^b) dx dz = \int g \left[ \int \partial _t (m^a-m^b) dx \right] dz = 0 \end{aligned}$$

since $\int \partial _t m^a dx = \int \partial _t m^b dx = 0$ for any *z*. Thus:

$$\begin{aligned}&\int \int (u^a-u^b) \partial _t (m^a-m^b) dx dz =\int \int (v^a-v^b) \partial _t (m^a-m^b) dx dz\\&\quad =\int \int (v^a-v^b) \mathcal {L}^*(m^a-m^b)dx dz + \int \int (v^a-v^b) (H_\nu (v^a) m^a-H_\nu (v^b)m^b) dx dz \end{aligned}$$

Finally, using the linearity of $\mathcal {L}$ and the definition of *g* above:

$$\begin{aligned}&\int \int (m^a-m^b) \mathcal {L}(u^a-u^b) dx dz -\int \int (m^a-m^b) \mathcal {L}(v^a-v^b) dx dz\\&=\int \int (m^a-m^b) \mathcal {L}(g) dx dz =\int \mathcal {L}(g) \left[ \int (m^a-m^b) dx\right] dz =0 \end{aligned}$$

since $\int m^a dx = \int m^b dx = n$ for any *z*.

Thus

$$\begin{aligned} \frac{d}{dt}\bar{K}(t)&= - \int \int (m^a-m^b) (F(m^a)-F(m^b)) dx dz \\&\quad - \int \int (m^a-m^b) (H(v^a)-H(v^b)) dx dz \\&\quad +\rho K(t) + \int \int (v^a-v^b) \mathcal {L}^*(m^a-m^b) dx dz\\&\quad - \int \int (m^a-m^b) \mathcal {L}(v^a-v^b) dx dz \\&\quad + \int \int (v^a-v^b)(H_\nu (v^a) m^a - H_\nu (v^b) m^b) dx dz \end{aligned}$$

$$\square $$

The next lemma analyzes two expressions of the previous lemma, using repeated integration by parts

### Lemma 5

Let $\{m^a,u^a,\bar{x}^a\}$ and $\{m^b,u^b,\bar{x}^b\}$ be two regular classical equilibria of the MFG. Then:

$$\begin{aligned}&\int _{-\infty }^{\infty }\int _{-\infty }^{\infty } \left( v^a(x,z,t) -v^b(x,z,t) \right) \mathcal {L}^*(m^a-m^b)(x,z,t) dx dz\\&\qquad -\int _{-\infty }^{\infty }\int _{-\infty }^{\infty } \left( m^a(x,z,t) -m^b(x,z,t) \right) \mathcal {L}(v^a-v^b)(x,z,t) dx dz \\&\quad \le \bar{H}_\nu \int _{-\infty }^{\infty } n(z)\, \left( v^b(\bar{x}^a(z,t),z,t) + v^a(\bar{x}^b(z,t),z,t) \right) dz. \end{aligned}$$

where $$\bar{H}_\nu \equiv \sup _{s,x,z} H_\nu (s,x,z)$$.

### Proof of Lemma 5

We omit the range of the integrals, which runs from $-\infty$ to $+\infty$ to simplify the notation. Let $\hat{v} = v^a-v^b$ and $\hat{m} = m^a-m^b$. Use the linearity of the operator to get:

$$\begin{aligned}&\int \int (v^a-v^b) \mathcal {L}^*(m^a-m^b)\, dx dz =\int \int \hat{v} \, \mathcal {L}^*(\hat{m})\, dx dz \\&\quad =-\int \int \hat{v} \, \partial _x ( \mu \hat{m}) \, dxdz - \int \int \hat{v} \, \partial _z (\hat{\mu }\hat{m})\, dzdx \\&\qquad +\int \int \hat{v} \, \partial _{xx}(\tfrac{1}{2} \sigma ^2 \hat{m}) \, dx dz + \int \int \hat{v} \, \partial _{zz}(\tfrac{1}{2} \hat{\sigma }^2 \hat{m})\, dz dx \end{aligned}$$

Likewise:

$$\begin{aligned}&\int \int (m^a-m^b)\mathcal {L}(v^a-v^b)\, dx dz =\int \int \hat{m} \mathcal {L}(\hat{v})\, dx dz \\&\quad =\int \int \hat{m} \mu \,\partial _x \hat{v} \, dx dz + \int \int \hat{m} \hat{\mu }\,\partial _z \hat{v} \, dz dx \\&\qquad +\int \int \hat{m} \tfrac{1}{2} \sigma ^2 \,\partial _{xx}\hat{v} \, dx dz + \int \int \hat{m} \tfrac{1}{2} \hat{\sigma }^2 \,\partial _{zz}\hat{v} \, dz dx \end{aligned}$$

Notice we have changed the order of integration across terms.

To compute $\int \int \hat{v} \mathcal {L}^*(\hat{m})dx dz$ we use that for a fixed *z* we integrate by parts with respect to *x* to obtain:

$$\begin{aligned}&-\int \hat{v} \, \partial _x ( \mu \hat{m}) \, dx = \int \mu \hat{m} \partial _x(\hat{v} ) \, dx - \hat{v} \mu \hat{m}|^{\infty }_{-\infty } \\&\int \hat{v} \, \partial _{xx}(\tfrac{1}{2} \sigma ^2 \hat{m}) \, dx =-\int \partial _x{\hat{v} }\, \partial _{x}(\tfrac{1}{2} \sigma ^2 \hat{m}) \, dx \\&\quad +\hat{v} \, \partial _{x}(\tfrac{1}{2} \sigma ^2 \hat{m})|_{-\infty }^{\bar{x}^a_-} +\hat{v} \, \partial _{x}(\tfrac{1}{2} \sigma ^2 \hat{m})|_{\bar{x}^a_+}^{\bar{x}^b_-} + \hat{v} \, \partial _{x}(\tfrac{1}{2} \sigma ^2 \hat{m})|_{\bar{x}^b_+}^{\infty } \end{aligned}$$

and for a fixed *x* we integrate by parts with respect to *z* to obtain:

$$\begin{aligned}&- \int \hat{v} \, \partial _z (\hat{\mu }\hat{m})\, dz = \int \hat{\mu }\hat{m}\, \partial _z \hat{v} \, dz - \hat{v} \hat{\mu }\hat{m}|^{\infty }_{-\infty } \\&\int \hat{m} \tfrac{1}{2} \hat{\sigma }^2 \,\partial _{zz}\hat{v} \, dz =- \int \partial _z(\hat{m} \tfrac{1}{2} \hat{\sigma }^2) \,\partial _{z}\hat{v} \, dz + \hat{m} \tfrac{1}{2} \hat{\sigma }^2 \,\partial _{z}\hat{v} |_{-\infty }^{\infty } \end{aligned}$$

Likewise to compute $-\int \int \hat{m} \, \mathcal {L}(\hat{v})\, dx dz$ we use that for a fixed *z* we integrate by parts with respect to *x* to obtain:

$$\begin{aligned}&-\int \hat{m} \mu \,\partial _x \hat{v} \, dx = \int \hat{v} \,\partial _x \hat{m} \mu \, dx - \hat{m} \mu \hat{v} |_{-\infty }^{\infty } \\&-\int \hat{m} \tfrac{1}{2} \sigma ^2 \,\partial _{xx}\hat{v} \, dx =\int \partial _x(\hat{m} \tfrac{1}{2} \sigma ^2) \,\partial _{x}\hat{v} \, dx -\hat{m} \tfrac{1}{2} \sigma ^2 \,\partial _{x}\hat{v} |_{-\infty }^{\infty } \end{aligned}$$

and for a fixed *x* we integrate by parts with respect to *x* to obtain:

$$\begin{aligned}&-\int \hat{m} \hat{\mu }\,\partial _z \hat{v} \, dz =\int \hat{v} \, \partial _z (\hat{m} \hat{\mu }) \, dz - \hat{m} \hat{\mu }\,\partial _z \hat{v} |_{-\infty }^{\infty } \\&- \int \hat{m} \tfrac{1}{2} \hat{\sigma }^2 \,\partial _{zz}\hat{v} \, dz =\int \partial _z(\hat{m} \tfrac{1}{2} \hat{\sigma }^2 ) \,\partial _{z}\hat{v} \, dz - \hat{m} \tfrac{1}{2} \hat{\sigma }^2 \,\partial _{z}\hat{v} |_{-\infty }^{\infty } \end{aligned}$$

Using the properties assumed for $\mu ,\sigma ^2$ and the integrability assumptions for $v^i$ and $m^i$ at a regular classical equilibrium we get that for a fixed *z*:

$$\begin{aligned} 0&=\hat{v} \mu \hat{m}|^{\infty }_{-\infty } =\hat{v} \, \partial _{x}(\tfrac{1}{2} \sigma ^2 \hat{m})|_{-\infty }^{\infty } =\hat{m} \mu \hat{v} |_{-\infty }^{\infty } =\hat{m} \tfrac{1}{2} \sigma ^2 \,\partial _{x}\hat{v} |_{-\infty }^{\infty } \end{aligned}$$

and likewise for a fixed *x* we get:

$$\begin{aligned} 0&= \hat{v} \hat{\mu }\hat{m}|^{\infty }_{-\infty } = \hat{m} \tfrac{1}{2} \hat{\sigma }^2 \,\partial _{z}\hat{v} |_{-\infty }^{\infty } = \hat{m} \hat{\mu }\,\partial _z \hat{v} |_{-\infty }^{\infty } = \hat{m} \tfrac{1}{2} \hat{\sigma }^2 \,\partial _{z}\hat{v} |_{-\infty }^{\infty } \end{aligned}$$

Note that

$$\begin{aligned} A&=\hat{v} \, \partial _{x}(\tfrac{1}{2} \sigma ^2 \hat{m})|_{-\infty }^{\bar{x}^a_-} +\hat{v} \, \partial _{x}(\tfrac{1}{2} \sigma ^2 \hat{m})|_{\bar{x}^a_+}^{\bar{x}^b_-} + \hat{v} \, \partial _{x}(\tfrac{1}{2} \sigma ^2 \hat{m})|_{\bar{x}^b_+}^{\infty } \\&=\hat{v} \, \partial _{x}(\tfrac{1}{2} \sigma ^2 \hat{m})|_{\bar{x}^a_+}^{\bar{x}^a_-} + \hat{v} \, \partial _{x}(\tfrac{1}{2} \sigma ^2 \hat{m})|_{\bar{x}^b_+}^{\bar{x}^b_-} \end{aligned}$$

Using that $\partial _x \sigma ^2(x,z)$ and *m*(*x*, *z*, *t*) are continuous on *x* everywhere we obtain:

$$\begin{aligned} A&\equiv \hat{v} \, \partial _{x}(\tfrac{1}{2} \sigma ^2 \hat{m})|_{\bar{x}^a_+}^{\bar{x}^a_-} + \hat{v} \, \partial _{x}(\tfrac{1}{2} \sigma ^2 \hat{m})|_{\bar{x}^b_+}^{\bar{x}^b_-} =\hat{v} \tfrac{1}{2} \sigma ^2 \, \partial _{x}\hat{m})|_{\bar{x}^a_+}^{\bar{x}^a_-} + \hat{v} \tfrac{1}{2} \sigma ^2 \, \partial _{x}\hat{m}|_{\bar{x}^b_+}^{\bar{x}^b_-} \end{aligned}$$

At this point it may be more clear to write all the arguments. In particular

$$\begin{aligned} A(z,t)&= \left( v^a(\bar{x}^a(z,t),z,t) - v^b(\bar{x}^a(z,t),z,t) \right) \tfrac{1}{2} \sigma ^2(\bar{x}^a(z,t),z) \\&\quad \times \left( \partial _x m^a(x,z,t) - \partial _x m^b(x,z,t) \right) |_{x=\bar{x}^a_+(z,t)}^{x=\bar{x}^a_-(z,t)} \\&\quad + \left( v^a(\bar{x}^b(z,t),z,t) - v^b(\bar{x}^b(z,t),z,t) \right) \tfrac{1}{2} \sigma ^2(\bar{x}^b(z,t),z) \\&\quad \times \left( \partial _x m^a(x,z,t) - \partial _x m^b(x,z,t) \right) |_{x=\bar{x}^b_+(z,t)}^{x=\bar{x}^b_-(z,t)} \end{aligned}$$

Using that $\partial _x m^a(x,z,t)$ is continuous at $\bar{x}^b(z,t)$ and that $\partial _x m^b(x,z,t)$ is continuous at $\bar{x}^a(z,t)$ whenever $\bar{x}^a(z,t)\ne \bar{x}^b(z,t)$. Also we use that $v^a(\bar{x}^a(z,t),z,t)=v^b(\bar{x}^b(z,t),z,t)=0$, then

$$\begin{aligned} A(z,t)&= - v^b(\bar{x}^a(z,t),z,t) \tfrac{1}{2} \sigma ^2(\bar{x}^a(z,t),z) \partial _x m^a(x,z,t) |_{x=\bar{x}^a_+(z,t)}^{x=\bar{x}^a_-(z,t)} \\&\quad - v^a(\bar{x}^b(z,t),z,t) \tfrac{1}{2} \sigma ^2(\bar{x}^b(z,t),z) \partial _x m^b(x,z,t) |_{x=\bar{x}^b_+(z,t)}^{x=\bar{x}^b_-(z,t)} \end{aligned}$$

And using Lemma 2, we have:

$$\begin{aligned} \tfrac{1}{2} \sigma ^2(\bar{x}^a(z,t),z) \partial _x m^a(x,z,t) |_{x=\bar{x}^a_+(z,t)}^{x=\bar{x}^a_-(z,t)}&=- \int _{-\infty }^{\infty } m^a(x,z,t) H_\nu ( v^a(x,z,t),x,z) \, dx \\&\ge - \bar{H}_\nu \int _{-\infty }^{\infty } m^a(x,z,t) dx= - \bar{H}_\nu \, n(z) \\ \tfrac{1}{2} \sigma ^2(\bar{x}^b(z,t),z) \partial _x m^b(x,z,t) |_{x=\bar{x}^b_+(z,t)}^{x=\bar{x}^b_-(z,t)}&=- \int _{-\infty }^{\infty } m^b(x,z,t) H_\nu ( v^a(x,z,t),x,z) \, dx \\&\ge - \bar{H}_\nu \int _{-\infty }^{\infty } m^b(x,z,t) dx = - \bar{H}_\nu \, n(z) \end{aligned}$$

where $\bar{H}_\nu$ is an upper bound on the derivative of $H_\nu$. Hence we have that:

$$\begin{aligned}&\int \int \hat{v} \mathcal {L}^*(\hat{m})dx dz -\int \int \hat{m} \, \mathcal {L}(\hat{v})\, dx dz \\&\quad \equiv \int _{-\infty }^{\infty }\int _{-\infty }^{\infty } \left( v^a(x,z,t) -v^b(x,z,t) \right) \mathcal {L}^*(m^a-m^b)(x,z,t) dx dz\\&\qquad -\int _{-\infty }^{\infty } \int _{-\infty }^{\infty } \left( m^a(x,z,t) -m^b(x,z,t) \right) \mathcal {L}(v^a-v^b)(x,z,t) dx dz \\&\quad \le \bar{H}_\nu \int _{-\infty }^{\infty } n(z)\, v^b(\bar{x}^a(z,t),z,t) dz +\bar{H}_\nu \int _{-\infty }^{\infty } n(z)\, v^a(\bar{x}^b(z,t),z,t) dz \end{aligned}$$

$$\square $$

### Proof of Proposition 2

The proof of this proposition follows directly by bounding each of the terms in decomposition of $\frac{d}{dt}\bar{K}(t)$ in Lemma 4. In particular, Lemma 3 shows that several integrals in $\frac{d}{dt}\bar{K}(t)$ can we written using $v^a-v^b$ instead of $u^a-u^b$. Lemma 5 shows that the terms in $\frac{d}{dt}\bar{K}(t)$ involving the integrals $\int (v^a-v^b)\mathcal {L}^*(m^a-m^b)$ and $-\int (m^a-m^b)\mathcal {L}(v^a-v^b)$ are both negative, so we write $\frac{d}{dt}\bar{K}(t)$ as an inequality. $\square$

### Proof of Proposition 3

The structure of the proof is similar to proof of Proposition 1. Below we will omit the domain of the integrals, which is $\mathbb {R}^n$, to simplify the notation. We note that the boundedness of *F* and $u_T$ implies that *u* and *v* are bounded. Since $v(x,t)=u(x,t) - \int u(z,t) \eta _\epsilon (z-\bar{x}(t))dz$ does not depend on *x*, then

$$\begin{aligned} \partial _x u(x,t) = \partial _x v(x,t),\quad \text {and}\quad \partial _{xx} u(x,t) = \partial _{xx} v(x,t) \end{aligned}$$

Using that $\int \left( m^a(x,t)-m^b(x,t)\right) dx=0$, then:

$$\begin{aligned}&\int \left( v^a(x,t)-v^b(x,t)\right) \left( m^a(x,t)-m^b(x,t)\right) dx \\&\quad =\int \left( u^a(x,t)-u^b(x,t)\right) \left( m^a(x,t)-m^b(x,t)\right) dx \end{aligned}$$

We start with:

$$\begin{aligned}&\frac{d}{dt}\int \left( u^a(x,t)-u^b(x,t)\right) \left( m^a(x,t)-m^b(x,t)\right) dx \\&\quad =\int \left( u^a(x,t)-u^b(x,t)\right) \partial _t \left( m^a(x,t)-m^b(x,t)\right) dx\\&\qquad +\int \left( m^a(x,t)-m^b(x,t)\right) \partial _t \left( u^a(x,t)-u^b(x,t)\right) dx \end{aligned}$$

Using the properties of $v^i(x,t)-u^i(x,t)=-u^i(\bar{x}^i(t),t)$ for $i=a,b$, and that $\int (m^a-m^b)dx$ and its time derivative are zero:

$$\begin{aligned}&\frac{d}{dt}\int \left( u^a(x,t)-u^b(x,t)\right) \left( m^a(x,t)-m^b(x,t)\right) dx \\&\quad =\int \left( u^a(x,t)-u^b(x,t)\right) \partial _t \left( m^a(x,t)-m^b(x,t)\right) dx\\&\qquad +\int \left( m^a(x,t)-m^b(x,t)\right) \partial _t \left( u^a(x,t)-u^b(x,t)\right) dx \\&\quad =\int \left( v^a(x,t)-v^b(x,t)\right) \partial _t \left( m^a(x,t)-m^b(x,t)\right) dx\\&\qquad +\int \left( m^a(x,t)-m^b(x,t)\right) \partial _t \left( u^a(x,t)-u^b(x,t)\right) dx \end{aligned}$$

Using the p.d.e for the HJB in (41) and the one in KBF (42) to replace in the previous integrals as follows:

$$\begin{aligned}&\frac{d}{dt}\int \left( u^a(x,t)-u^b(x,t)\right) \left( m^a(x,t)-m^b(x,t)\right) dx \\&\quad =\int \left( v^a-v^b\right) \left( \mathcal {L}^*(m^a-m^b) \right) - \left( m^a-m^b\right) \left( \mathcal {L}(u^a-u^b) \right) \\&\qquad +\rho \int \left( u^a-u^b\right) \left( m^a-m^b\right) dx\\&\qquad + \int \left( v^a-v^b\right) \left( m^a H_\nu \left( v^a\right) - m^b H_\nu \left( v^b \right) \right) dx \\&\qquad - \int \left( m^a-m^b\right) \left( H\left( v^a\right) - H\left( v^b \right) \right) dx \\&\qquad - \int \left( m^a-m^b\right) \left( F(x,m^a)- F(x,m^b)\right) dx \\&\qquad - \int \left( v^a-v^b\right) \left( \left( \int H_\nu \left( v^a\right) m^a \right) \eta ^a- \left( \int H_\nu \left( v^b\right) m^b\right) \eta ^b \right) dx \end{aligned}$$

where we omit the arguments (*x*, *t*) or *x* from the different functions to simplify the notation, and where we use the notation:

$$\begin{aligned} H\left( v^i \right)&\text { as } H\left( u(x,t) - \int u^i(z,t)\eta _\epsilon (z-\bar{x}^i(t)) dz , x\right) \\ H_\nu \left( v^i \right)&\text { as } H_\nu \left( u(x,t) - \int u^i(z,t)\eta _\epsilon (z-\bar{x}^i(t)) dz , x\right) \\ v^i&\text { as } u(x,t) - \int u^i(z,t)\eta _\epsilon (z-\bar{x}^i(t)) dz \\ \eta ^i&\text { as } \eta _\epsilon (z-\bar{x}^i(t)) \end{aligned}$$

for $i=a,b$. Using that $\partial _x v=\partial _x u$ and $\partial _{xx} v=\partial _{xx} u$ we can write:

$$\begin{aligned}&\frac{d}{dt}\int \left( u^a(x,t)-u^b(x,t)\right) \left( m^a(x,t)-m^b(x,t)\right) dx \\&\quad =\int \left( v^a-v^b\right) \left( \mathcal {L}^*(m^a-m^b) \right) - \left( m^a-m^b\right) \left( \mathcal {L}(v^a-v^b) \right) dx\\&\qquad +\rho \int \left( u^a-u^b\right) \left( m^a-m^b\right) dx\\&\qquad + \int \left( v^a-v^b\right) \left( m^a H_\nu \left( v^a\right) - m^b H_\nu \left( v^b \right) \right) dx \\&\qquad - \int \left( m^a-m^b\right) \left( H\left( v^a \right) - H\left( v^b \right) \right) dx \\&\qquad - \int \left( m^a-m^b\right) \left( F(x,m^a)- F(x,m^b)\right) dx\\&\qquad - \int \left( v^a-v^b\right) \left( \left( \int H_\nu \left( v^a\right) m^a \right) \eta ^a- \left( \int H_\nu \left( v^b\right) m^b\right) \eta ^b \right) dx \end{aligned}$$

Thus we write:

$$\begin{aligned} \frac{d}{dt}\int \left( u^a-u^b\right) \left( m^a-m^b\right) dx&=\rho \int \left( u^a-u^b\right) \left( m^a-m^b\right) dx \\&+ I_{L}(t)+I_H(t) +I_F (t) \end{aligned}$$

where

$$\begin{aligned} I_{L}(t)&\equiv \int \left( v^a-v^b\right) \left( \mathcal {L}^*(m^a-m^b) \right) - \left( m^a-m^b\right) \left( \mathcal {L}(v^a-v^b) \right) dx \\ I_F(t)&\equiv - \int \left( m^a-m^b\right) \left( F(x,m^a) - F(x,m^b)\right) dx \\ I_H(t)&\equiv \int \left( v^a-v^b\right) \left( m^a H_\nu \left( v^a\right) - m^b H_\nu \left( v^b \right) \right) dx \\&\quad - \int \left( m^a-m^b\right) \left( H\left( v^a \right) - H\left( v^b \right) \right) dx \\&\quad - \int \left( v^a-v^b\right) \left( \left( \int H_\nu \left( v^a\right) m^a \right) \eta ^a- \left( \int H_\nu \left( v^b\right) m^b\right) \eta ^b \right) dx \end{aligned}$$

Next, we obtain an inequality from each of the following terms.

1. We have that
  $$\begin{aligned} I_L(t) =0 \end{aligned}$$
  since $\mathcal {L}$ and $\mathcal {L}^*$ are adjoints. The boundary terms do not contribute given the assumption in (a).
2. $I_F(t)\le 0$: holds directly by the assumption of weak monotonicity of *F*, i.e., that
  $$\int \left( m^a-m^b\right) \left( F(x,m^a) - F(x,m^b)\right) dx\ge 0.$$
3. We will show that:
  $$\begin{aligned} I_H(t)&\le \int \left( v^a-v^b\right) \left( m^a H_\nu \left( v^a\right) - m^b H_\nu \left( v^b \right) \right) dx \\&- \int \left( m^a-m^b\right) \left( H\left( v^a \right) - H\left( v^b \right) \right) dx \end{aligned}$$
  This follows from expanding each term, i.e.,
  $$\begin{aligned} I_H(t) \equiv&\int \left( v^a-v^b\right) \left( m^a H_\nu \left( v^a\right) - m^b H_\nu \left( v^b \right) \right) dx \\&- \int \left( m^a-m^b\right) \left( H\left( v^a \right) - H\left( v^b \right) \right) dx \\&- \int \left( v^a-v^b\right) \left( \left( \int H_\nu \left( v^a\right) m^a \right) \eta ^a- \left( \int H_\nu \left( v^b\right) m^b\right) \eta ^b \right) dx \end{aligned}$$
  We can rewrite the last line to get:
  $$\begin{aligned} I_H(t) =&\int \left[ m^a + m^b \right] \frac{\bar{H}_{\nu \nu }(v^a,v^b)}{2}\left( v^a- v^b\right) ^2 dx \\&- \left( \int H_\nu \left( v^a\right) m^a dx \right) \int \left( v^a-v^b\right) \eta ^a dx \\&- \left( \int H_\nu \left( v^b\right) m^b dx \right) \int \left( v^b-v^a\right) \eta ^b dx \end{aligned}$$
  Using the definition we have:
  $$\begin{aligned} \int v^a\eta ^a dx =&\int \left[ u^a(x,t) - \int u^a(z,t) \eta _\epsilon (z-\bar{x}^a(t) ) dz \right] \eta _\epsilon (x-\bar{x}^a(t) ) dx\\ =&\int u^a(x,t) \eta _\epsilon (x-\bar{x}^a(t)) dx - \int u^a(z,t) \eta _\epsilon (z-\bar{x}^a(t)) dz =0 \end{aligned}$$
  and
  $$\begin{aligned} \int v^b\eta ^a dx =&\int \left[ v^b(x,t) \right] \eta _\epsilon (x-\bar{x}^a(t) ) dx \ge 0 \end{aligned}$$
  Thus:
  $$\begin{aligned} \int \left( v^a-v^b\right) \eta ^a dx \le 0 \text { and } \int \left( v^b-v^a\right) \eta ^b dx \le 0 \end{aligned}$$
  and using that
  $$\begin{aligned} - \left( \int H_\nu \left( v^a\right) m^a dx \right) \ge 0 \text { and } - \left( \int H_\nu \left( v^b\right) m^b dx \right) \ge 0 \end{aligned}$$
  Hence we obtain the desired result.

Combining the expressions for $I_{L}(t)$, $I_{H}(t)$, $I_F(t)$ we obtain the desired result for the proposition. $\square$

## Discrete time version of the decision problem

In this section we define a discrete time version of the continuous time problems analyzed in Sects. 3.1 and 3.2. We first define the control problem of the decision maker in sequence form and the corresponding Bellman equation. Our discrete time notation is chosen so that the corresponding continuous time formulation is immediate. Finally we use the discrete time Bellman equation to formally derive the continuous time HJB equation for each model, namely (10) and (15) used in the body of the paper.

### Costly probability model

We let $\Delta$ be the length of the time period. We assume that when *x* is uncontrolled it evolves as:

$$\begin{aligned} x_{t+\Delta } = x_t + \mu (x_t) \Delta + \sigma (x_t) \sqrt{\Delta } \, e_{t+\Delta } \end{aligned}$$

where

$$\begin{aligned} e_{t+\Delta } = {\left\{ \begin{array}{ll} +1 &{} \text { with probability} = \frac{1}{2}\\ -1 &{} \text { with probability} = \frac{1}{2} \end{array}\right. } \end{aligned}$$

and where $e_{t+\Delta }$ is independent of $e_r$ for any *r*.

To simplify notation, we let $f(x,t) = F(x,m(t))$, and a discount factor given by $1/(1+\Delta \rho )$. Let us consider an agent with a value *x* at time *t*, before any decisions are made, so the agent gets $f(x,t) \Delta$ flow cost during the discrete time period *t* to $t+\Delta$.

We consider a Poisson point process $N_t$, that counts the number of adjustment opportunities that the agent had until period *t*. In particular if $N_{t}-N_{t-\Delta }=1$ the agent has an adjustment opportunity. Otherwise the agent cannot adjust and *x* is uncontrolled. In particular we assume

$$\begin{aligned} N_{t}-N_{t-\Delta } = {\left\{ \begin{array}{ll} 1 &{} \text { with probability} = \lambda (x_t,t) \Delta \\ 0 &{} \text { with probability} = 1- \lambda (x_t,t) \Delta \end{array}\right. } \end{aligned}$$

for some function $\lambda (x_t,t)$ that is chosen by the decision maker as part of the policy. In other words the agent decides the probability $\lambda$ per unit of time of an adjustment opportunity. The agent pays a cost $c(x,\lambda )\Delta$ if she chooses $\lambda$.

*The control problem.* The policy *p* of the decision maker consists of choosing two objects: the first is the probability per unit of time, $\lambda (x,t)$ of an adjustment opportunity at every *t* and *x*. The second is $\bar{x}(t)$, which is the value of *x* at which the state is set if the adjustment opportunity occurs. Hence $\lambda : \mathbb R \times [0,\Delta ,2\Delta ,\ldots ,T-\Delta ]\rightarrow [0,\frac{1}{\Delta }]$ and $\bar{x}: [\Delta ,2\Delta ,\ldots ,T]\rightarrow \mathbb R$. To simplify notation we restrict the policy to depend only on *x* and *t*, which by the principle of optimality is without loss of generality. For a given policy the law of motion of *x* is

$$\begin{aligned} x^p_{t+\Delta } - x^p_t = {\left\{ \begin{array}{ll} \mu (x^p_t) \Delta + \sigma (x^p_t) \sqrt{\Delta } &{} \text { with Prob. } \ \ \frac{1}{2} (1-\Delta \lambda (x^p_t,t) ) \\ \mu (x^p_t) \Delta - \sigma (x^p_t) \sqrt{\Delta } &{} \text { with Prob. }\ \ \frac{1}{2} (1-\Delta \lambda (x^p_t,t) ) \\ \bar{x}_{t+\Delta } - x^p_t &{} \text { with Prob. }\ \ \Delta \lambda (x^p_t,t) \end{array}\right. } \end{aligned}$$

for $t=0,\Delta ,\ldots ,T-\Delta$. The value for the decision maker of a given policy, and the stochastic process generated by it, is

$$\begin{aligned} J^p(x,t)&=\mathbb E \Bigg [\sum _{r=t, \ldots , T-\Delta } \, (1+\rho \Delta )^{-\frac{r-t}{\Delta }} \Big ( f(x^p_r,r) + c(x^p_r,\lambda (x^p_r,r)) \Big ) \Delta \\&\quad +(1+\rho \Delta )^{-\frac{T-t}{\Delta }} u_T(x^p_T,m(T)) \Big | x_t=x \Bigg ] \end{aligned}$$

where the expectation is taken with respect to the process for $x^p$ for all $t=0,\Delta ,\ldots , T-\Delta$ and *x*.

*Bellman equation. * We denote the value function by $u(x,t)=\min _p J^p(x,t)$. Before writing down the discrete time Bellman equation, we introduce the following notation for the conditional expectation of the next period value function:

$$\begin{aligned} E_x(u_{t+\Delta }) \equiv \frac{1}{2} u\left( x+ \mu (x) \Delta + \sigma (x) \sqrt{\Delta } , t+\Delta \right) +\frac{1}{2} u\left( x+ \mu (x) \Delta - \sigma (x) \sqrt{\Delta } , t+\Delta \right) \end{aligned}$$

We can now write the discrete time Bellman equation for this problem:

$$\begin{aligned} u(x,t)&= f(x,t) \Delta + \min _{\{ 0\le \lambda \le 1/\Delta \}} c(x,\lambda ) \Delta \\&\quad + \frac{1}{1+\rho \Delta } (1-\lambda \Delta ) E_x(u_{t+\Delta }) + \frac{1}{1+\rho \Delta } \lambda \Delta u(\bar{x}_{t+\Delta },t+\Delta ) \\ \text { where } \bar{x}_{t+\Delta }&= \arg \min _y u(y,t+\Delta ) \end{aligned}$$

The first term of the right hand side is the period cost. The second term has the cost of selecting $\lambda$. The third and fourth terms correspond to the continuation. The third term corresponds to the case where the agent does not get the opportunity to adjust. The last one contains the case where the agent has a possibility of adjusting the state.

*Heuristic derivation of the continuous time HJB equation. * Multiplying both sides by $(1+\rho \Delta )$

$$\begin{aligned} (1+\rho \Delta )u(x,t)&= f(x,t) (1+\rho \Delta ) \Delta + \min _{\{ 0\le \lambda \le 1/\Delta \}} c(x,\lambda ) (1+\rho \Delta ) \Delta \\&\quad + (1-\lambda \Delta ) E_x(u_{t+\Delta }) + \lambda \Delta u(\bar{x}_{t+\Delta },t+\Delta ) \end{aligned}$$

rearranging

$$\begin{aligned} \rho \Delta u(x,t)&= f(x,t) (1+\rho \Delta ) \Delta + (1+\rho \Delta )\min _{\{ 0\le \lambda \le 1/\Delta \}} c(x,\lambda ) (1+\rho \Delta ) \Delta \\&\quad + E_x(u_{t+\Delta }) - u(x,t) + \lambda \Delta \left[ u(\bar{x}_{t+\Delta },t+\Delta ) - E_x(u_{t+\Delta }) \right] \end{aligned}$$

Dividing by $$\Delta $$

$$\begin{aligned} \rho u(x,t)&= f(x,t) (1+\rho \Delta ) + (1+\rho \Delta )\min _{\{ 0\le \lambda \le 1/\Delta \}} c(x,\lambda ) (1+\rho \Delta ) \\&\quad + \frac{E_x(u_{t+\Delta }) - u(x,t)}{\Delta } + \lambda \left[ u(\bar{x}_{t+\Delta },t+\Delta ) - E_x(u_{t+\Delta }) \right] \end{aligned}$$

Taking $\Delta \downarrow 0$ and using that:

$$\begin{aligned} \frac{ E_x(u_{t+\Delta })- u(x,t) }{\Delta }&\rightarrow u_x(x,t) \mu (x) + \frac{\sigma ^2(x)}{2}u_{xx}(x,t)+ u_t(x,t) \\ E_x(u_{t+\Delta })&\rightarrow u(x,t) \\ u(\bar{x}_{t+\Delta },t+\Delta )&\rightarrow u(\bar{x}(t),t) \\ f(x,t) (1+\rho \Delta )&\rightarrow f(x,t) \end{aligned}$$

We obtain the desired result:

$$\begin{aligned} \rho u(x,t)&= f(x,t) + \min _{\{ 0\le \lambda \}} c(x,\lambda ) + \lambda \left[ u(\bar{x}(t),t) - u(x,t) \right] \\&\quad +u_x(x,t) \mu (x) + \frac{\sigma ^2(x)}{2}u_{xx}(x,t) + u_t(x,t) \end{aligned}$$

### Random fixed cost model

We let $\Delta$ be the length of the time period. We assume that when *x* is uncontrolled it evolves as:

$$\begin{aligned} x_{t+\Delta } = x_t + \mu (x_t) \Delta + \sigma (x_t) \sqrt{\Delta } \, e_{t+\Delta } \end{aligned}$$

where

$$\begin{aligned} e_{t+\Delta } = {\left\{ \begin{array}{ll} +1 &{} \text { with probability} = \frac{1}{2}\\ -1 &{} \text { with probability} = \frac{1}{2} \end{array}\right. } \end{aligned}$$

and where $e_{t+\Delta }$ is independent of $e_r$ for any time *r*. We consider a Poisson point process $N_t$, that counts the number of adjustment opportunities that the agent had until period *t*. In particular if $N_{t}-N_{t-\Delta }=1$ the agent has an adjustment opportunity. Otherwise the agent cannot adjust and *x* is uncontrolled. In particular we assume

$$\begin{aligned} N_{t}-N_{t-\Delta } = {\left\{ \begin{array}{ll} 1 &{} \text { with probability} = \kappa (x_t) \Delta \\ 0 &{} \text { with probability} = 1- \kappa (x_t) \Delta \end{array}\right. } \end{aligned}$$

for some function $\kappa$ that is given to the decision maker.

When an adjustment opportunity occurs the agent draws a fixed cost $\psi$ from a distribution with CDF $G(\psi )$. If the agent pays $\psi$ then she can adjust *x* to any value. We label the realizations of the cost drawn up to time *t* as $\{\psi _0, \psi _1,\ldots , \psi _{N_t} \}$. The realizations of $N_{r+\Delta }-N_{r}$, $\psi _r$ are independent of the realizations of $e_{r'}$ for all $r,r'$.

To simplify notation, we let $f(x,t) = F(x,(m(t))$ and a discount factor given by $1/(1+\Delta \rho )$. Let us consider an agent with a value *x* at time *t*, before any decisions are made, so the agent gets $f(x,t) \Delta$ flow cost during the discrete time period *t* to $t+\Delta$. Without loss of generality, we consider policies described by a threshold *s* for each *x*, *t*: the agent pays $\psi$ and adjusts only if $\psi _{N_t} \le s(x,t)$.

*The control problem.* The policy *p* of the decision maker consists of choosing two objects: the first is the threshold *s*(*x*, *t*) for the fixed cost that triggers an adjustment conditional on $N_{t}-N_{t-\Delta } =1$. The second is $\bar{x}(t)$, the value of *x* at which the state is set after the adjustment. Hence $s: \mathbb R \times [0,\Delta ,2\Delta ,\ldots ,T-\Delta ]\rightarrow \mathbb R_+$ and $\bar{x}: [\Delta ,2\Delta ,\ldots ,T]\rightarrow \mathbb R$. To simplify notation we restrict the policy to depend only on *x* and *t*, which by the principle of optimality is without loss of generality. For a given policy the law of motion of *x* is

$$\begin{aligned} x^p_{t+\Delta } - x^p_t = {\left\{ \begin{array}{ll} \mu (x^p_t) \Delta + \sigma (x^p_t) \sqrt{\Delta } &{} \text { with Prob. } \ \ \frac{1}{2} \left( 1-\Delta \kappa (x^p_t) \textbf{1}_{\{ \psi _{N_t} \le s(x^p_t,t) \}} \right) \\ \mu (x^p_t) \Delta - \sigma (x^p_t) \sqrt{\Delta } &{} \text { with Prob. }\ \ \frac{1}{2} \left( 1-\Delta \kappa (x^p_t) \textbf{1}_{\{ \psi _{N_t} \le s(x^p_t,t) \}} \right) \\ \bar{x}_{t+\Delta } - x^p_t &{} \text { with Prob. }\ \ \Delta \kappa (x^p_t) \textbf{1}_{\{ \psi _{N_t} \le s(x^p_t,t) \}} \end{array}\right. } \end{aligned}$$

for $t=0,\Delta ,\ldots ,T-\Delta$. The value for the decision maker of a given policy, and the stochastic process generated by it, is

$$\begin{aligned} J^p(x,t)&=\mathbb E \Big [\sum _{r=t, \ldots , T-\Delta } \, (1+\rho \Delta )^{-\frac{r-t}{\Delta }} \Big ( f(x^p_r,r) \Delta + \psi _{N_r} \, \textbf{1}_{\{ \psi _{N_r} \le s(x^p_r,r) \}} \Big )\\&\quad + (1+\rho \Delta )^{-\frac{T-t}{\Delta }} u_T(x^p_T,m(T)) \Big | x_t=x \Big ] \end{aligned}$$

where the expectation is taken with respect to the process for $x^p$ for all $t=0,\Delta ,\ldots , T-\Delta$ and *x*.

*Bellman equation. * We denote the value function by $u(x,t)=\min _p J^p(x,t)$. Before writing down the discrete time Bellman equation, we introduce the following notation for the conditional expectation of the next period value function:

$$\begin{aligned} E_x(u_{t+\Delta }) \equiv \frac{1}{2} u\left( x+ \mu (x) \Delta + \sigma (x) \sqrt{\Delta } , t+\Delta \right) +\frac{1}{2} u\left( x+ \mu (x) \Delta - \sigma (x) \sqrt{\Delta } , t+\Delta \right) \end{aligned}$$

We can now write the discrete time Bellman equation for this problem:

$$\begin{aligned} u(x,t)&= f(x,t) \Delta + \frac{1}{1+\rho \Delta } (1-\kappa (x)\Delta ) E_x(u_{t+\Delta }) \\&\quad + \frac{1}{1+\rho \Delta } \kappa (x)\Delta \int \min \left\{ E_x(u_{t+\Delta }) \, , \, \psi + u(\bar{x}_{t+\Delta },t+\Delta ) \right\} dG(\psi ) \\ \text { where } \bar{x}_{t+\Delta }&= \arg \min _y u(y,t+\Delta ) \end{aligned}$$

The first term of the right hand side is the period cost. The second and third term correspond to the continuation. The second term corresponds to the case where the agent does not get the opportunity to adjust. The last one contains the case where the agent has a possibility of adjusting the state. This last term contains the only decision of the problem.

*Heuristic derivation of the continuous time HJB equation.* Multiplying both sides by $(1+\rho \Delta )$

$$\begin{aligned} (1+\rho \Delta )u(x,t)&= f(x,t) (1+\rho \Delta ) \Delta + (1-\kappa (x)\Delta ) E_x(u_{t+\Delta }) \\&\quad + \kappa (x)\Delta \int \min \left\{ E_x(u_{t+\Delta }) \, , \, \psi + u(\bar{x}_{t+\Delta },t+\Delta ) \right\} dG(\psi ) \end{aligned}$$

rearranging

$$\begin{aligned} u(x,t)\rho \Delta&= f(x,t) (1+\rho \Delta ) \Delta + E_x(u_{t+\Delta })- u(x,t) \\&\quad + \kappa (x)\Delta \left[ \int \min \left\{ E_x(u_{t+\Delta }) \, , \, \psi + u(\bar{x}_{t+\Delta },t+\Delta ) \right\} dG(\psi ) - E_x(u_{t+\Delta }) \right] \end{aligned}$$

collecting terms in the minimum:

$$\begin{aligned} u(x,t)\rho \Delta&= f(x,t) (1+\rho \Delta ) \Delta + E_x(u_{t+\Delta })- u(x,t) \\&\quad + \kappa (x)\Delta \int \min \left\{ 0 \, , \, \psi + u(\bar{x}_{t+\Delta },t+\Delta )- E_x(u_{t+\Delta }) \right\} dG(\psi ) \end{aligned}$$

Dividing by $$\Delta $$

$$\begin{aligned} u(x,t)\rho&= f(x,t) (1+\rho \Delta ) + \frac{ E_x(u_{t+\Delta })- u(x,t) }{\Delta }\\&\quad + \kappa (x) \int \min \left\{ 0 \, , \, \psi + u(\bar{x}_{t+\Delta },t+\Delta )- E_x(u_{t+\Delta }) \right\} dG(\psi ) \end{aligned}$$

Taking $\Delta \downarrow 0$ and using that:

$$\begin{aligned} \frac{ E_x(u_{t+\Delta })- u(x,t) }{\Delta }&\rightarrow u_x(x,t) \mu (x) + \frac{\sigma ^2(x)}{2}u_{xx}(x,t)+ u_t(x,t) \\ E_x(u_{t+\Delta })&\rightarrow u(x,t) \\ u(\bar{x}_{t+\Delta },t+\Delta )&\rightarrow u(\bar{x}(t),t) \\ f(x,t) (1+\rho \Delta )&\rightarrow f(x,t) \end{aligned}$$

We obtain the desired result:

$$\begin{aligned} \rho u(x,t)&= f(x,t) + u_x(x,t) \mu (x) + \frac{\sigma ^2(x)}{2}u_{xx}(x,t)+ u_t(x,t)\\&\quad + \kappa (x) \int \min \left\{ 0 \, , \, \psi + u(\bar{x}(t),t)- u(x,t) \right\} dG(\psi ) \end{aligned}$$

## Footnotes

1 As mentioned, a key aspect of the MFG concept is that the decision maker’s flow cost function depends on the cross-sectional distribution of the state.

2 We also consider the more general case where the agent draws two costs, one for increases and one for decreases.

3 Menaldi and Robin [20] considers a more general specifications for the distribution of the random times where the decision maker can control the state, and a different specification of the adjustment cost.

4 In Appendix B we give a detailed description of the control problem and we derive the corresponding HJB formulation used in the MFG.

5 See Caceres et al. [15] for an application with a similar term.
